# Vamp3/syntaxin 4 mediates the basolateral membrane fusion of TfR transcytosis across the BBB and is exploited by pathogenic *Escherichia coli*

**DOI:** 10.1073/pnas.2500285122

**Published:** 2025-07-02

**Authors:** Bin Liu, Yingying Su, Hao Sun, Bin Yang, Lili Wan, Xiaoya Li, Shaobin Hou, Guozhen Ma, Juan Joanna Yu, Lu Feng, Huamin Henry Li, Lei Wang

**Affiliations:** ^a^National Key Laboratory of Intelligent Tracking and Forecasting for Infectious Diseases, TEDA Institute of Biological Sciences and Biotechnology, Nankai University, Tianjin 300457, China; ^b^The Key Laboratory of Molecular Microbiology and Technology, Ministry of Education, Nankai University, Tianjin 300071, China; ^c^Nankai International Advanced Research Institute, Nankai University Shenzhen, Shenzhen 518045, China; ^d^Institute for Asthma and Allergy, Chevy Chase, MD 20815; ^e^Southwest United Graduate School, Kunming 650092, China

**Keywords:** blood-brain barrier, transferrin receptor transcytosis, neonatal meningitis *Escherichia coli*

## Abstract

Transcytosis across the blood–brain barrier (BBB), composed of brain microvascular endothelial cells (HBMECs), is tightly controlled to prevent the entry of macromolecules, microorganisms, and toxins into the brain. Transferrin receptor (TfR) transcytosis, one of the few active transcytosis pathways in HBMECs, is extensively utilized for drug transport across the BBB and employed by meningitis-causing bacteria to penetrate into the brain. However, the molecular mechanism facilitating the fusion of TfR vesicles with the basolateral membrane of HBMECs, the final step of transcytosis, has not been experimentally studied. Here, we found that the interaction between the v-SNARE protein VAMP3 on TfR vesicles and the t-SNARE protein syntaxin 4 that is limited to the basolateral membrane in HBMECs mediates the fusion. We also provided evidence that this step is critical for the efficiency of TfR transcytosis. Furthermore, we showed that neonatal meningitis *Escherichia coli* (NMEC) infection significantly boosts the efficiency of this transcytosis by enhancing the expression of VAMP3 and syntaxin 4 through the TLR4-TRAM-TRIF-TRAF3-IKK-IRF3 signaling pathway in HBMECs. Silence or overexpression of VAMP3 and syntaxin 4 reduces or enhances the transcytosis of transferrin and NMEC in a human BBB model *in vitro*. Consistently, the penetration of transferrin and NMEC into the brain was significantly inhibited in VAMP3-deficient mice. These findings provide insights for improving strategies to deliver drugs into the brain and developing effective therapy for meningitis caused by NMEC.

## Significance

Transferrin receptor (TfR) transcytosis is an essential strategy for delivering therapeutics into the brain and is exploited by meningitis-causing bacteria to penetrate the blood–brain barrier (BBB). We found that the interaction between VAMP3 on TfR vesicles and syntaxin 4 at the basolateral membrane of brain microvascular endothelial cells mediates the final fusion step of TfR transcytosis. Enhancing the expression of VAMP3 and syntaxin 4 promotes TfR transcytosis across the BBB. Furthermore, neonatal meningitis *Escherichia coli* (NMEC) increases its transcytosis efficiency by upregulating host VAMP3 and syntaxin 4 expression through the LPS-TLR4 signaling pathway. Our finding suggests that modulating the expression of VAMP3 and syntaxin 4 could be an effective strategy to improve brain drug delivery or to treat NMEC infection.

The BBB, which is composed of human brain microvascular endothelial cells (HBMECs), is an essential gatekeeper for the central nervous system (CNS), uniquely separating brain internal milieu from the circulating blood (1). With the feature of numerous intercellular tight junctions and low rates of transcytosis, the BBB is characterized by a very low permeability for biomolecules, microorganisms, and toxins in order to protect and regulate the metabolism of the brain and maintain the neural microenvironment (2, 3).

To guarantee the proper functioning of the brain, several low-rate transcytosis pathways across the BBB are employed under tight regulation to ensure an adequate supply of ions, nutrients, and essential signaling molecules required by nervous tissue (4). Among these pathways, transcytosis mediated by the transferrin receptor (TfR) provides an important route for delivering iron to the brain, which is essential for multiple neurological functions (5, 6). The impermeable nature of the BBB poses a significant challenge to the uptake of therapeutic agents into the brain(7). The TfR transcytosis-mediated delivery system has been extensively utilized for the transport of drugs across the BBB (8, 9) and is considered one of the most promising brain delivery approaches (4). Several TfR-targeting antibodies and antibody–drug conjugates have demonstrated encouraging outcomes for brain delivery in clinical trials (8, 10, 11). However, despite significant efforts have been made to improve the TfR transcytosis-mediated delivery system through methods such as antibody engineering (10, 12, 13), its efficiency remains relatively low. Recently, we found that three major meningitis-causing bacterial pathogens with different evolutionary history, including neonatal meningitis *Escherichia coli* (NMEC), *Streptococcus pneumoniae,* and group B *Streptococcus*, which invade the brain from the bloodstream to cause diseases, have employed the same mechanism to cross the BBB. They all activate the same fusion process of bacteria-containing vesicles (BCVs) with TfR vesicles within HBMECs, thereby hijacking TfR transcytosis to penetrate the BBB (14). This finding further demonstrates that utilizing TfR transcytosis for the transport of drugs across the BBB is the correct option.

For TfR transcytosis across the BBB, holo-Tf (Tf-Fe) first binds to the TfR at the apical membrane (blood side) of HBMECs, and the resulting Tf–TfR complex enters the cell as vesicles through clathrin-mediated endocytosis, forming TfR vesicles (15). For approximately 90% of the endocytosed Tf–TfR complex, the iron is released from Tf in the cytoplasm (15), and this portion of the Tf–TfR complex does not reach the basolateral membrane of HBMECs. The rest of the endocytosed holo-Tf traffics to the basolateral membrane (brain side) of HBMECs. These vesicles then fuse with the basolateral membrane of HBMECs, which is critical for the ultimate release of holo-Tf into the brain (6). However, the mechanism governing this process remains unclear. We hypothesized that increasing the fusion of TfR vesicles with the basolateral membrane of HBMECs, based on understanding the mechanism underlying this membrane fusion process, can enhance the efficiency of TfR transcytosis across the BBB.

SNARE proteins, divided into v-SNAREs on vesicles and t-SNAREs on target membranes, are the principal elements responsible for membrane fusion in different cells (16). In nonpolarized CHO cells and HeLa cells, the v-SNARE protein VAMP2 or VAMP3 present on TfR vesicles have been found to mediate the fusion of TfR vesicles with plasma membrane that lacks distinct polarity, respectively, contributing to the exocytic event of recycling TfR vesicles(17–19). It remains unclear which SNARE proteins in polarized HBMECs are involved in the fusion between TfR vesicles and the basolateral membrane.

In this study, we first revealed that VAMP3 and syntaxin 4 are located at the TfR vesicles and the basolateral membrane of HBMECs, respectively, and their interactions directly mediate the transcytosis of Tf and NMEC. Silence or mutation of VAMP3 and syntaxin 4 reduced the transcytosis of Tf and NMEC across the BBB in vitro and in mice. We found that NMEC infection induces VAMP3 and syntaxin 4 expression, via the activation of the TLR4-TRAM-TRIF-TRAF3-IKK-IRF3 regulatory pathway by LPS. Furthermore, we showed that high overexpression of VAMP3 or syntaxin 4 promotes the fusion of TfR vesicles and TfR-NMEC vesicles with the basolateral membrane of HBMECs, thus increasing the efficiency of TfR-mediated transcytosis. This work provides essential insights for developing strategies for the treatment of meningitis caused by NMEC and improving drug delivery into the brain.

## Results

### VAMP3 Contributes to the Transcytosis of Tf Across the BBB.

**Fig. 1.**
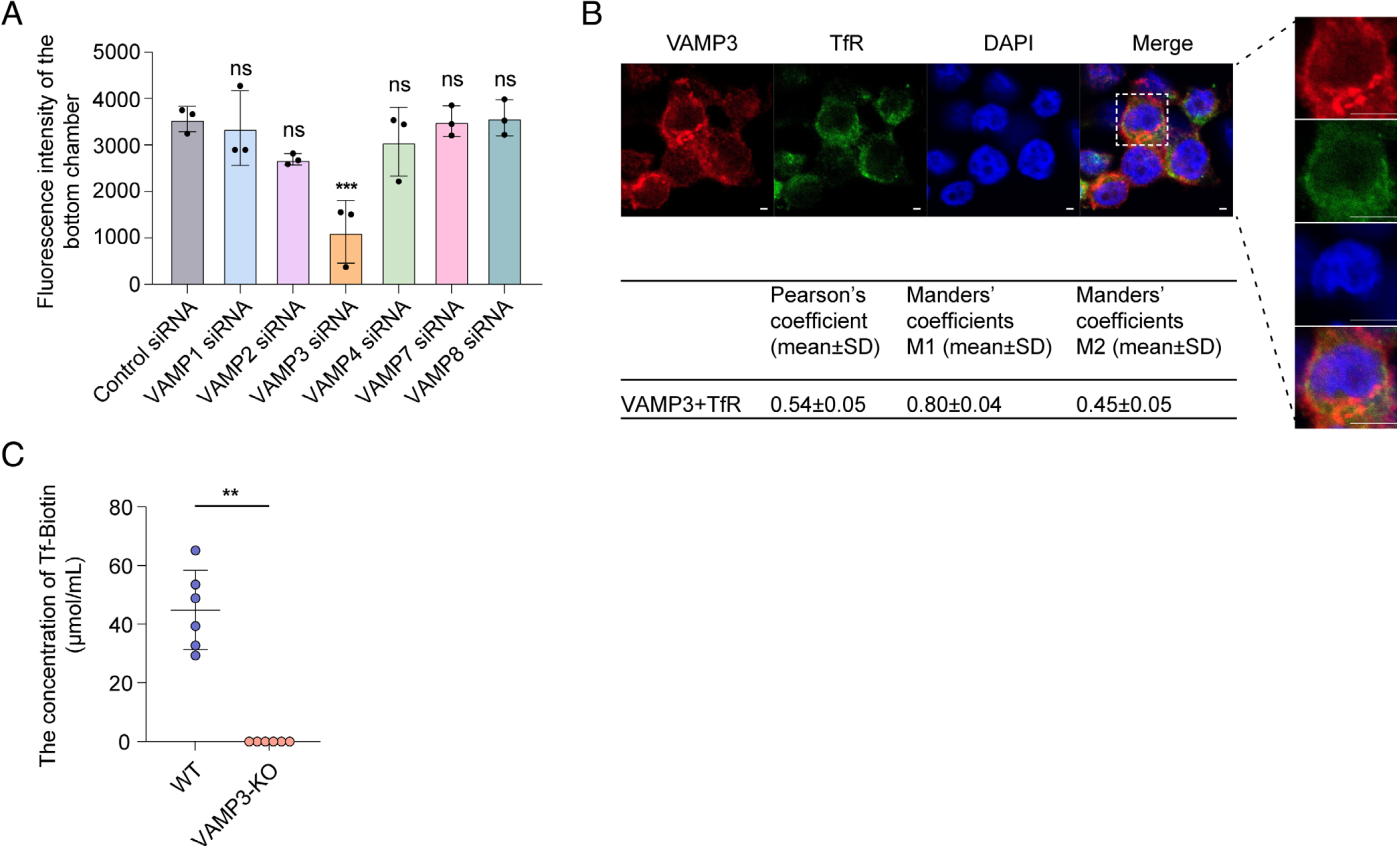
VAMP3 is involved in the transcytosis of Tf through HBMECs. (*A*) Spectrophotometric measurement of fluorescence intensity to assess FITC-Tf transcytosis from the apical side to the basolateral side of HBMECs transfected with control siRNA or siRNA targeting VAMP1, VAMP2, VAMP3, VAMP4, VAMP7, and VAMP8. The excitation is 495 nm, and the emission is 518 nm. (n = 3 independent experiments). (*B*) Colocalization of VAMP3 (red) with TfR (green) in HBMECs. Cell nuclei were stained with DAPI (blue). Quantification of colocalization was shown by calculating the Pearson correlation coefficients (PC), Mander’s coefficient M1 (fraction of VAMP3 colocalized with TfR), and Mander’s coefficient M2 (fraction of TfR colocalized with VAMP3). n = 5 random areas per group from three independent experiments. (Scale bar, 2 μm.) (*C*) Effect of VAMP3 deficiency on transcytosis of Tf–biotin across the BBB in vivo. WT (wild type) mice and gene-deficient VAMP3^−/−^ (VAMP3 KO) mice (n = 6) received Tf–biotin via the tail vein. 1 μL cerebrospinal fluid from each mouse was collected after 4 h for kit detection. Data are presented as the means ± SD, *P < 0.05; **P < 0.01; ***P < 0.001, ns, nonsignificant. One-way ANOVA (*A*) and Mann–Whitney *U* test (*C*).

VAMP1, VAMP2, VAMP3, VAMP4, VAMP7, and VAMP8 are major v-SNARE proteins involved in the membrane fusion step of the exocytosis process of different cells (17, 20, 21). We first investigated whether some of these v-SNARE proteins are involved in the transcytosis of Tf through HBMECs, using a polarized HBMEC monolayer with tight junctions in Transwells in vitro as described previously(14). We showed that silencing VAMP3 of HBMECs with siRNA (reduction by 90%, *SI Appendix*, Fig. S1) significantly reduced the amount of FITC-Tf transcytosed from the apical side to the basolateral side of the cell (Fig. 1*A*); however, silencing VAMP1, VAMP2, VAMP4, VAMP7, or VAMP8 by siRNA (reduction by 90 to 96%, *SI Appendix*, Fig. S1) did not affect the transcytosis of FITC-Tf across the HBMEC monolayer (Fig. 1*A*). Colocalization of VAMP3 with TfR was observed in HBMECs using confocal microscopy (Fig. 1*B*). The Pearson’s correlation and Manders’ overlap coefficients were used to measure the colocalization of VAMP3 with TfR (Pearson’s coefficient was 0.54; Manders’ coefficient M1 (the fraction of VAMP3 colocalized with TfR) was 0.80, and M2 (the fraction of TfR colocalized with VAMP3) was 0.45). It indicates that within HBMECs, the majority of VAMP3 is located on TfR vesicles, whereas only a fraction of TfR vesicles carry VAMP3. Furthermore, we investigated whether VAMP3 influences the penetration of Tf across the BBB in vivo. We showed that gene-deficient VAMP3^−/−^ mice exhibited a significantly reduced amount of Tf–biotin in the cerebrospinal fluid (CSF) after tail vein injection compared to wild-type mice (Fig. 1*C*), indicating that the transcytosis of Tf–biotin across the BBB of VAMP3^−/−^ mice was significantly inhibited. These data indicate that VAMP3 present on the TfR vesicles promotes the transcytosis of Tf across the BBB in vitro and in vivo.

### The Interaction of VAMP3 with Syntaxin 4 at the Basolateral Membrane Mediates the Final Fusion Step of TfR Transcytosis Across HBMECs.

**Fig. 2.**
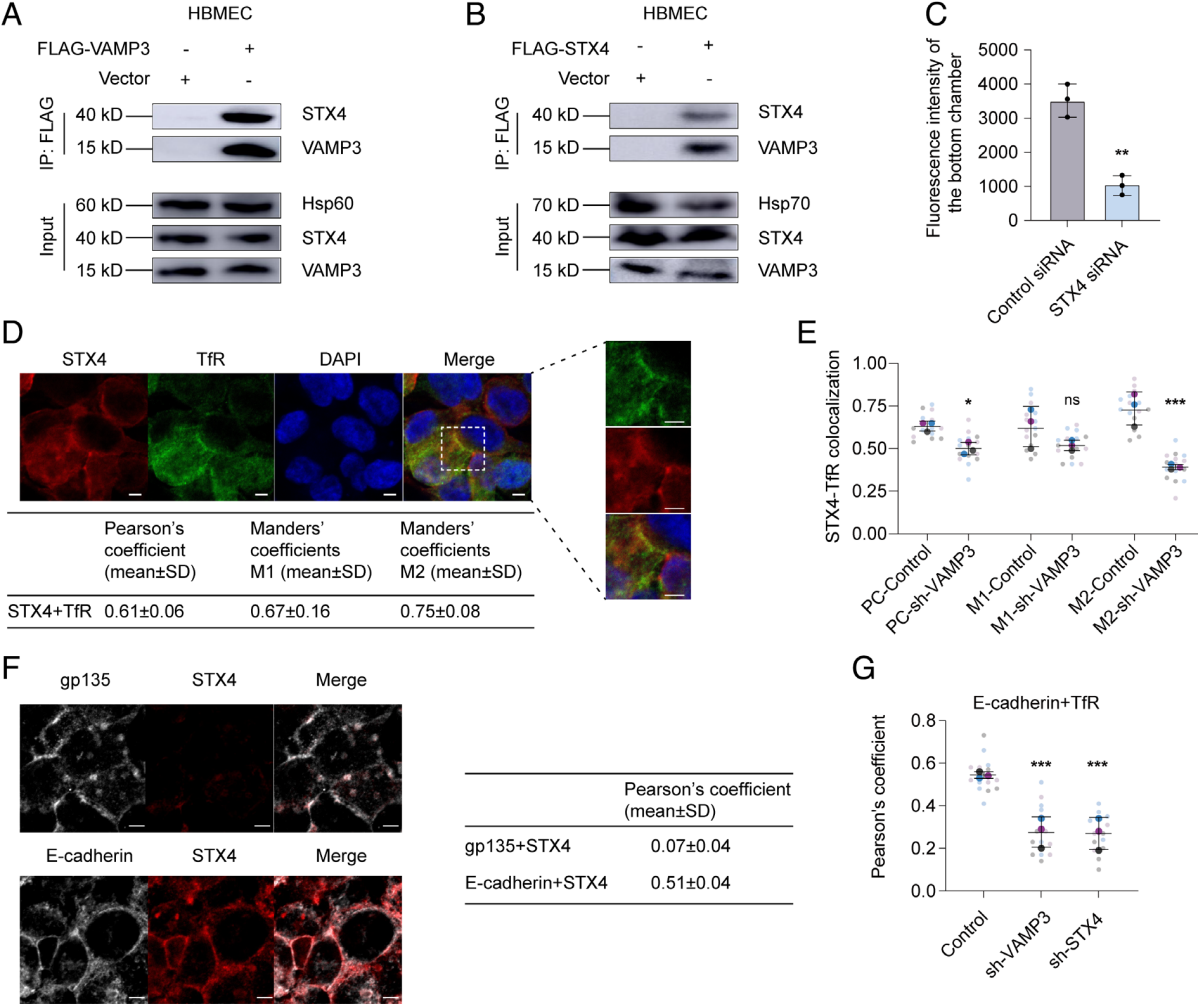
VAMP3 interacts with syntaxin 4 (STX4) at the basolateral membrane of HBMECs. (*A* and *B*) Coimmunoprecipitation assays of VAMP3 and STX4 in HBMECs. Hsp60 and Hsp70, loading control. (n = 3 independent experiments). (*C*) Spectrophotometric measurement of fluorescence intensity to assess FITC-Tf transcytosis from the apical side to the basolateral side of HBMECs transfected with control siRNA or siRNA targeting STX4. (n = 3 independent experiments). (*D* and *E*) Colocalization of STX4 (red) with TfR (green) in polarized HBMECs (*D*), polarized HBMECs transfected with the pGMLV-SC5 lentiviral vector harboring shRNA targeting VAMP3 (sh-VAMP3) or control shRNA (*E*). Cell nuclei were stained with DAPI (blue). (Scale bar, 2 μm.) Quantification of colocalization was shown by calculating the Pearson correlation coefficients (PC), Mander’s coefficient M1 (fraction of STX4 colocalized with TfR), and Mander’s coefficient M2 (fraction of TfR colocalized with STX4). (*F*) Colocalization of STX4 (red) with gp135 (white, *Top*) or E-cadherin (white, *Bottom*) in polarized HBMECs, respectively. Quantification of colocalization was shown by calculating the Pearson correlation coefficients (PC). (Scale bar, 2 μm.) (*G*) Colocalization of E-cadherin with TfR in polarized HBMECs transfected with the pGMLV-SC5 lentiviral vector harboring shRNA targeting VAMP3, STX4, or control shRNA. Quantification of colocalization was shown by calculating the Pearson correlation coefficients (PC). The immunofluorescence (IF) data are quantified using Superplot (*E* and *G*), which concisely visualizes individual data points and their averages. The distinct combinations of colors indicate the three independent experiments performed. Horizontal lines show mean ± SD. Each small dot in the graph corresponds to a specific data point representing an analyzed image. The larger dots represent the average values calculated from the respective data points. Data are shown as the mean ± SD, *P < 0.05, **P < 0.01, ***P < 0.001, ns, nonsignificant. Two-tailed unpaired Student’s *t* test (*C*), two-way ANOVA (*E*), and one-way ANOVA (*G*).

VAMP3 has been reported to interact with t-SNARE syntaxin 4 to mediate the membrane fusion events in several different cells (21–23). Hence, we hypothesized that VAMP3 on TfR vesicles may also interact with syntaxin 4 in HBMECs. To test this, we carried out coimmunoprecipitation assays using HBMECs transfected with FLAG-tagged VAMP3 or FLAG-tagged syntaxin 4. The results showed that VAMP3 was efficiently coimmunoprecipitated with syntaxin 4 (Fig. 2 *A* and *B*), indicating that there is interaction between VAMP3 and syntaxin 4 within HBMECs. As a control, we showed that syntaxin 18 was not coimmunoprecipitated with VAMP3 or syntaxin 4 (*SI Appendix*, Fig. S2 *A* and *B*). Next, we investigated whether syntaxin 4 also contributes to the transcytosis of Tf across HBMECs. We showed that silencing syntaxin 4 (reduction by 97%, *SI Appendix*, Fig. S2*C*) significantly reduced the amount of FITC-Tf transcytosed from the apical side to the basolateral side of polarized HBMEC monolayer in Transwells (Fig. 2*C*). As a control, silencing syntaxin 3 (reduction by 57%, *SI Appendix*, Fig. S2*D*) did not affect the transcytosis of FITC-Tf across the HBMEC monolayer (*SI Appendix*, Fig. S2*E*). Using confocal microscopy, colocalization of syntaxin 4 with TfR was observed in polarized HBMECs (Fig. 2*D*, Pearson’s coefficient was 0.61, Manders’ coefficient M1 was 0.67, and Manders’ coefficient M2 was 0.75). Their colocalization was decreased in HBMECs with stable VAMP3-knockdown using lentivirus compared with control cells (Fig. 2*E*), indicating that the colocalization of syntaxin 4 with TfR is dependent on VAMP3. Collectively, these data indicate that syntaxin 4 interactions with VAMP3 also play a role in the transcytosis of Tf across HBMECs.

The residency of syntaxin 4 is reported to be limited to the basolateral membrane in MDCK (Madin-Darby canine kidney) cells, a polarized epithelial cell line (24). We next confirmed that syntaxin 4 is also located at the basolateral membrane of polarized HBMECs. Confocal microscopy analysis was used to analyze the location of syntaxin 4 in polarized HBMEC monolayer in Transwells, with the basolateral membrane marker E-cadherin and apical plasma membrane marker gp135. Only the colocalization of syntaxin 4 with E-cadherin was observed in polarized HBMECs (Pearson’s coefficient was 0.51), and no intracellular staining of syntaxin 4 was detected (Fig. 2*F*). It indicates that syntaxin 4 that interacts with VAMP3 is located at the basolateral membrane of HBMECs.

Furthermore, using confocal microscopy, we showed that there was a significant decrease in the colocalization of TfR with the basolateral membrane of polarized HBMECs with stable VAMP3 or syntaxin 4-knockdown using lentivirus compared with control cells (Fig. 2*G*), indicating that a decrease in the fusion of TfR vesicles with the basolateral membrane when VAMP3 or syntaxin 4 was silenced. As a control, we showed that stable knockdown of syntaxin 3, which is reported to be limited to the apical membrane in MDCK cells (24), did not influence the colocalization of TfR with the basolateral membrane of HBMECs (*SI Appendix*, Fig. S2*F*). Collectively, these data indicate that the interaction between VAMP3 on TfR vesicles and syntaxin 4 at the basolateral membrane of HBMECs, mediating the fusion of TfR vesicles with the basolateral membrane, is essential for the transcytosis of Tf across HBMECs.

It has been reported that the t-SNARE SNAP23 interacts with VAMP3 and syntaxin 4 to form a complex that mediates membrane fusion in several cells (22, 23, 25). To investigate whether SNAP23 also contributes to the transcytosis of Tf through HBMECs, we performed a Transwell assay and demonstrated that silencing SNAP23 (reduction by 58%, *SI Appendix*, Fig. S2*G*) significantly reduced the amount of FITC-Tf transcytosed from the apical side to the basolateral side of polarized HBMEC monolayer in Transwells (*SI Appendix*, Fig. S2*H*). Furthermore, coimmunoprecipitation assays showed that VAMP3 and syntaxin 4 efficiently coimmunoprecipitated with SNAP23 in HBMECs (*SI Appendix*, Fig. S2*I*). These data suggest that SNAP23 probably plays a role in the transcytosis of Tf across HBMECs by interacting with VAMP3 and syntaxin 4.

### Overexpression of VAMP3 and Syntaxin 4 Enhances the Efficiency of TfR Transcytosis.

**Fig. 3.**
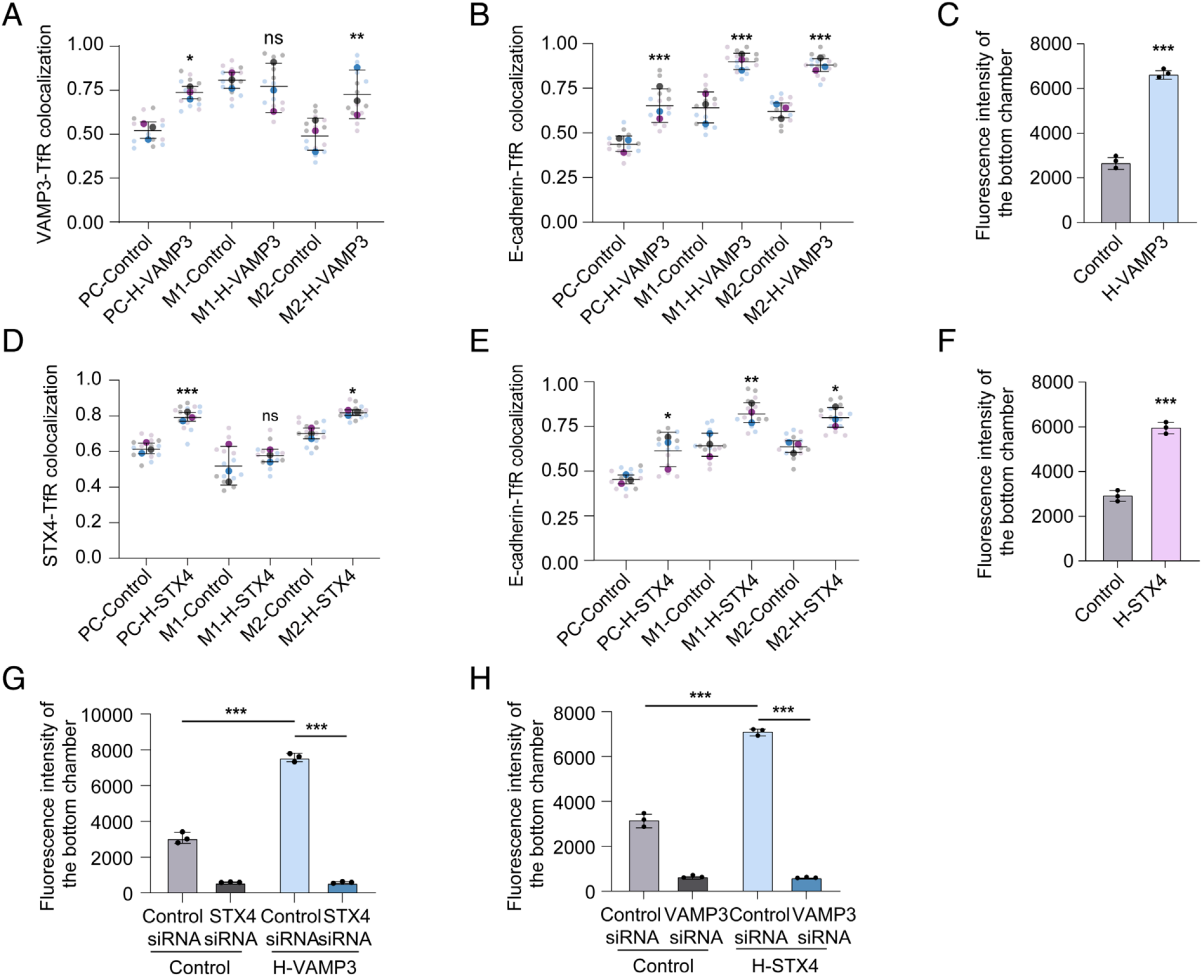
Overexpression of VAMP3 and syntaxin 4 (STX4) enhances the efficiency of TfR transcytosis. (*A*) Colocalization of VAMP3 with TfR in polarized VAMP3-overexpressing (H-VAMP3) cells and control cells. Quantification of colocalization was shown by calculating the Pearson correlation coefficients (PC), Mander’s coefficient M1 (fraction of VAMP3 colocalized with TfR), and Mander’s coefficient M2 (fraction of TfR colocalized with VAMP3). (*B*) Colocalization of E-cadherin with TfR in polarized H-VAMP3 cells and control cells. Quantification of colocalization was shown by calculating the Pearson correlation coefficients (PC), Mander’s coefficient M1 (fraction of E-cadherin colocalized with TfR), and Mander’s coefficient M2 (fraction of TfR colocalized with E-cadherin). (*C*) Spectrophotometric measurement of fluorescence intensity to assess FITC-Tf transcytosis from the apical side to the basolateral side of H-VAMP3 cells and control cells. n = 3 independent experiments. (*D*) Colocalization of STX4 with TfR in polarized STX4-overexpressing (H-STX4) cells and control cells. Quantification of colocalization was shown by calculating the Pearson correlation coefficients (PC), Mander’s coefficient M1 (fraction of STX4 colocalized with TfR), and Mander’s coefficient M2 (fraction of TfR colocalized with STX4). (*E*) Colocalization of E-cadherin with TfR in polarized H-STX4 cells and control cells. Quantification of colocalization was shown by calculating the Pearson correlation coefficients (PC), Mander’s coefficient M1 (fraction of E-cadherin colocalized with TfR), and Mander’s coefficient M2 (fraction of TfR colocalized with E-cadherin). (*F*) Spectrophotometric measurement of fluorescence intensity to assess FITC-Tf transcytosis from the apical side to the basolateral side of H-STX4 cells and control cells. n = 3 independent experiments. (*G* and *H*) Spectrophotometric measurement of fluorescence intensity to assess FITC-Tf transcytosis from the apical side to the basolateral side of control cells compared with H-VAMP3 cells (*G*) or H-STX4 cells (H) transfected with control siRNA and STX4 siRNA (*G*), or VAMP3 siRNA (H). n = 3 independent experiments. The IF data are quantified using Superplot (*A*, *B*, *D*, and *E*), which concisely visualizes individual data points and their averages. The distinct combinations of colors indicate the three independent experiments performed. Horizontal lines show mean ± SD. Each small dot in the graph corresponds to a specific data point representing an analyzed image. The larger dots represent the average values calculated from the respective data points. Data are shown as the mean ± SD, *P < 0.05, **P < 0.01, ***P < 0.001, ns, nonsignificant. Two-way ANOVA (*A*, *B*, *D*, *E*, *G*, and *H*) and two-tailed unpaired Student’s *t* test (*C* and *F*).

As the above results showed that only a fraction of TfR vesicles within HBMECs are positive for VAMP3 (Manders’ coefficient M2 was 0.45) (Fig. 1*B*), we hypothesized that increasing the proportion of TfR vesicles carrying VAMP3 by overexpressing VAMP3 can promote the fusion of TfR vesicles with the basolateral membrane, thereby enhancing the transcytosis of Tf across HBMECs. To test this hypothesis, we stably overexpressed VAMP3 in HBMECs. Confocal microscopy showed that in HBMECs stably overexpressing VAMP3, the proportion of TfR carrying VAMP3 increased compared with control cells (M2 increased from 0.50 to 0.72) (Fig. 3*A*). Furthermore, the colocalization of TfR with the basolateral membrane was also increased in HBMECs overexpressing VAMP3 (M2, the fraction of TfR colocalized with E-cadherin, increased from 0.62 to 0.87) (Fig. 3*B*). Additionally, we showed that the efficiency of Tf transcytosis through HBMECs stably overexpressing VAMP3 was enhanced by 2.46-fold compared with control cells (Fig. 3*C*). These data indicate that increasing VAMP3 expression enhances the proportion of TfR vesicles carrying VAMP3, leading to increased fusion between TfR vesicles and the basolateral membrane and, consequently, a higher efficiency of TfR transcytosis.

As VAMP3 interacts with syntaxin 4 to mediate the fusion of TfR vesicles with the basolateral membrane, we hypothesized that overexpressing syntaxin 4 also can promote the transcytosis of Tf across HBMECs. Confocal microscopy showed that in HBMECs stably overexpressing syntaxin 4 (syntaxin 4 was also only present at the basolateral membrane, *SI Appendix*, Fig. S3*A*), the proportion of TfR overlapping syntaxin 4 was increased compared with control cells (M2 increased from 0.72 to 0.81) (Fig. 3*D*). In addition, the colocalization of TfR with the basolateral membrane was also increased in HBMECs overexpressing syntaxin 4 (M2 increased from 0.65 to 0.79) (Fig. 3*E*). However, stable overexpression of syntaxin 4 had no effect on the colocalization of VAMP3 with TfR (*SI Appendix*, Fig. S3*B*), indicating that syntaxin 4 does not influence the recruitment of VAMP3 to TfR vesicles. Consistently, the transcytosis of Tf through HBMECs stably overexpressing syntaxin 4 was enhanced by 2.02-fold compared with control cells (Fig. 3*F*). However, we showed that silencing VAMP3 or syntaxin 4 inhibited the beneficial effect of overexpressing syntaxin 4 or VAMP3, respectively, on Tf transcytosis (Fig. 3 *G* and *H*). It is consistent with that VAMP3 and syntaxin 4 function collaboratively to mediate the TfR transcytosis across HBMECs. As a control, we showed that stable overexpression of syntaxin 3 in HBMECs had no effect on the colocalization of TfR with the basolateral membrane or on the transcytosis of Tf through HBMECs (*SI Appendix*, Fig. S3 *C* and *D*). Additionally, we demonstrated that silencing or overexpressing VAMP3 or syntaxin 4 in HBMECs had no effect on the colocalization of TfR with the apical membrane (*SI Appendix*, Fig. S3 *E* and *F*), indicating that VAMP3 and syntaxin 4 do not influence the recycling of TfR vesicles within HBMECs. Collectively, these data indicate that overexpression of VAMP3 and syntaxin 4 promotes the fusion of TfR vesicles with the basolateral membrane, thus increasing the efficiency of TfR transcytosis across HBMECs.

### VAMP3 and Syntaxin 4 Contribute to the Transcytosis of NMEC.

**Fig. 4.**
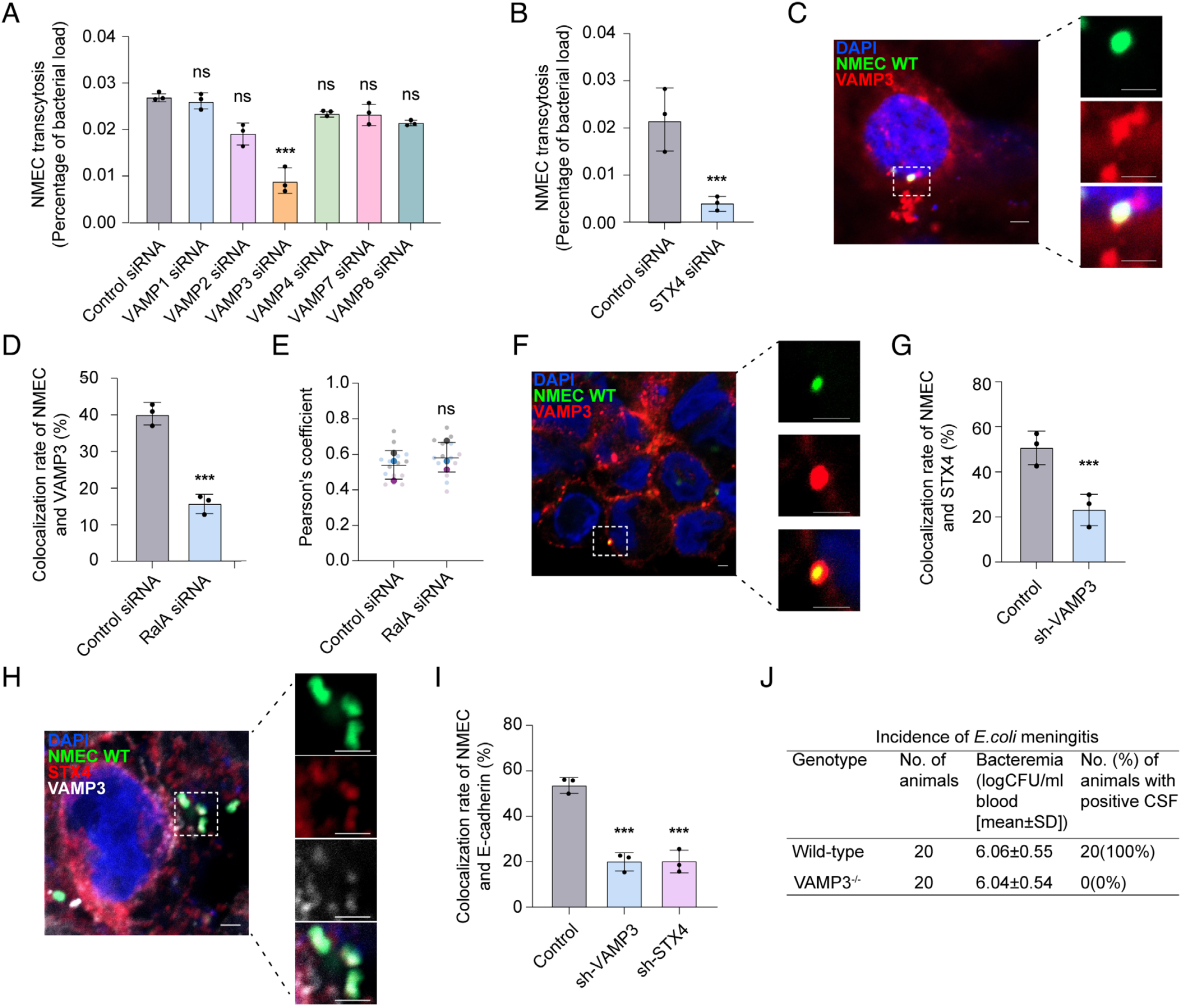
VAMP3 and syntaxin 4 (STX4) contribute to the transcytosis of NMEC. (*A* and *B*) Transcytosis of NMEC across HBMECs transfected with control siRNA, VAMP1, VAMP2, VAMP3, VAMP4, VAMP7, VAMP8 siRNA (*A*) or STX4 siRNA (*B*). (*C* and *D*) Colocalization of intracellular NMEC with VAMP3 in infected HBMECs (*C*) or HBMECs transfected with control siRNA or RalA siRNA (*D*). The numbers indicate the percentage of BCVs colocalized with VAMP3 relative to total intracellular BCVs (n = 3 slides). (Scale bar, 5 μm.) (*E*) Colocalization of VAMP3 with TfR in infected HBMECs transfected with control siRNA or RalA siRNA. Quantification of colocalization was shown by calculating the Pearson correlation coefficients (PC). The IF data are quantified using Superplot, which concisely visualizes individual data points and their averages. The distinct combinations of colors indicate the three independent experiments performed. Horizontal lines show mean ± SD. Each small dot in the graph corresponds to a specific data point representing an analyzed image. The larger dots represent the average values calculated from the respective data points. (*F*) Colocalization of STX4 (red) with the intracellular NMEC (green) in polarized HBMECs. (Scale bar, 5 μm.) (*G*) Colocalization of intracellular NMEC with STX4 in polarized HBMECs transfected with the pGMLV-SC5 lentiviral vector harboring shRNA targeting VAMP3 or control shRNA. The numbers indicate the percentage of BCVs colocalized with STX4 relative to total intracellular BCVs (n = 3 slides). (*H*) Colocalization of STX4 (red), VAMP3 (white), and intracellular NMEC (green) in polarized HBMECs. (Scale bar, 5 μm.) (*I*) Colocalization of intracellular NMEC with E-cadherin in polarized HBMECs transfected with the pGMLV-SC5 lentiviral vector harboring shRNA targeting VAMP3, STX4, or control shRNA. The numbers indicate the percentage of BCVs colocalized with E-cadherin relative to total intracellular BCVs (n = 3 slides). (*J*) The effect of VAMP3 on penetration of the BBB by NMEC in vivo. Eighteen-day-old C57BL/6N mice and VAMP3^−/−^ mice (n = 20) were infected with NMEC via the tail vein and killed at 4 h p.i. The blood samples were collected for bacteremia measurement, and the CSF was collected and cultured to indicate the incidence of meningitis. The positive CSF cultures were defined as meningitis. n = 3 independent experiments. Data are shown as the mean ± SD, *P < 0.05, **P < 0.01, ***P < 0.001, ns, nonsignificant. One-way ANOVA (*A* and *I*) and two-tailed unpaired Student’s *t* test (*B*, *D*, *E*, and *G*).

Next, as our recent work showed that NMEC couple its penetration of BBB to TfR transcytosis (14), we investigated whether VAMP3 on TfR vesicles and syntaxin 4 at the basolateral membrane of HBMECs also contributes to the transcytosis of NMEC. We showed that silencing VAMP3 or syntaxin 4 significantly reduced the transcytosis of NMEC across the polarized HBMEC monolayer in Transwells in vitro (Fig. 4 *A* and *B*). In contrast, silencing VAMP3 or syntaxin 4 had no effect on the bacterial invasion of HBMECs (*SI Appendix*, Fig. S4*A*) and the fusion of BCVs with TfR vesicles within HBMECs (*SI Appendix*, Fig. S4*B*). As a control, silencing syntaxin 3 did not affect the transcytosis of NMEC across the HBMEC monolayer (*SI Appendix*, Fig. S4*C*). It indicates that VAMP3 and syntaxin 4 contribute to the transcytosis of NMEC across HBMECs.

Confocal microscopy showed that 40.3% BCVs were colocalized with VAMP3 (Fig. 4*C*). As the above results showed that VAMP3 is present on TfR vesicles in HBMECs, we investigated whether the colocalization of VAMP3 with BCVs depends on the fusion of BCVs and TfR vesicles. We showed that silencing RalA (reduction by 88%, *SI Appendix*, Fig. S4*D*), which is known to decrease the fusion of BCVs and TfR vesicles (14), significantly reduced the colocalization of VAMP3 with BCVs (Fig. 4*D*). In contrast, silencing RalA did not influence the colocalization of VAMP3 with TfR (Fig. 4*E*). In addition, we showed that inhibition of protein synthesis in HBMECs prior to NMEC infection had no effect on the colocalization of VAMP3 with BCVs (*SI Appendix*, Fig. S4*E*). These data indicate that VAMP3 colocalized with BCVs originates from TfR vesicles in HBMECs.

Furthermore, confocal microscopy showed that 51.2% of BCVs were colocalized with syntaxin 4 in polarized HBMECs (Fig. 4*F*). We found that there was a significant decrease in colocalization between BCVs and syntaxin 4 in polarized HBMECs with stable VAMP3-knockdown compared with control cells (Fig. 4*G*), indicating that the interaction between BCVs and syntaxin 4 depends on VAMP3. Consistently, the colocalization of VAMP3, syntaxin 4, and BCVs was observed at the basolateral membrane of polarized HBMECs (Fig. 4*H*). Furthermore, we observed that the percent of NMEC colocalized with the basolateral region of infected HBMECs with stable VAMP3-knockdown or syntaxin 4-knockdown exhibited a significant decrease compared with control cells (Fig. 4*I*). As a control, stable knockdown of syntaxin 3 did not affect the colonization of BCVs with the basolateral region of infected HBMECs (*SI Appendix*, Fig. S4*F*). It indicates that VAMP3 and syntaxin 4 mediate the fusion of TfR-NMEC vesicles with the basolateral membrane of HBMECs. As the disruption of syntaxin 4 results in early embryonic lethality of mice (26), we used gene-deficient VAMP3^−/−^ mice to perform the animal experiments. The results showed that VAMP3^−/−^ mice exhibited significantly reduced bacterial titers in the cerebrospinal fluid after tail vein infection compared to wild-type mice (Fig. 4*J*), indicating the transcytosis of NMEC across the BBB was inhibited in VAMP3^−/−^ mice. Collectively, these data indicate that VAMP3 and syntaxin 4 contribute to the transcytosis of NMEC across the BBB by mediating the fusion of TfR-NMEC vesicles with the basolateral membrane of HBMECs.

### NMEC Increases Its Transcytosis Efficiency by Enhancing the Expression of VAMP3 and Syntaxin 4.

**Fig. 5.**
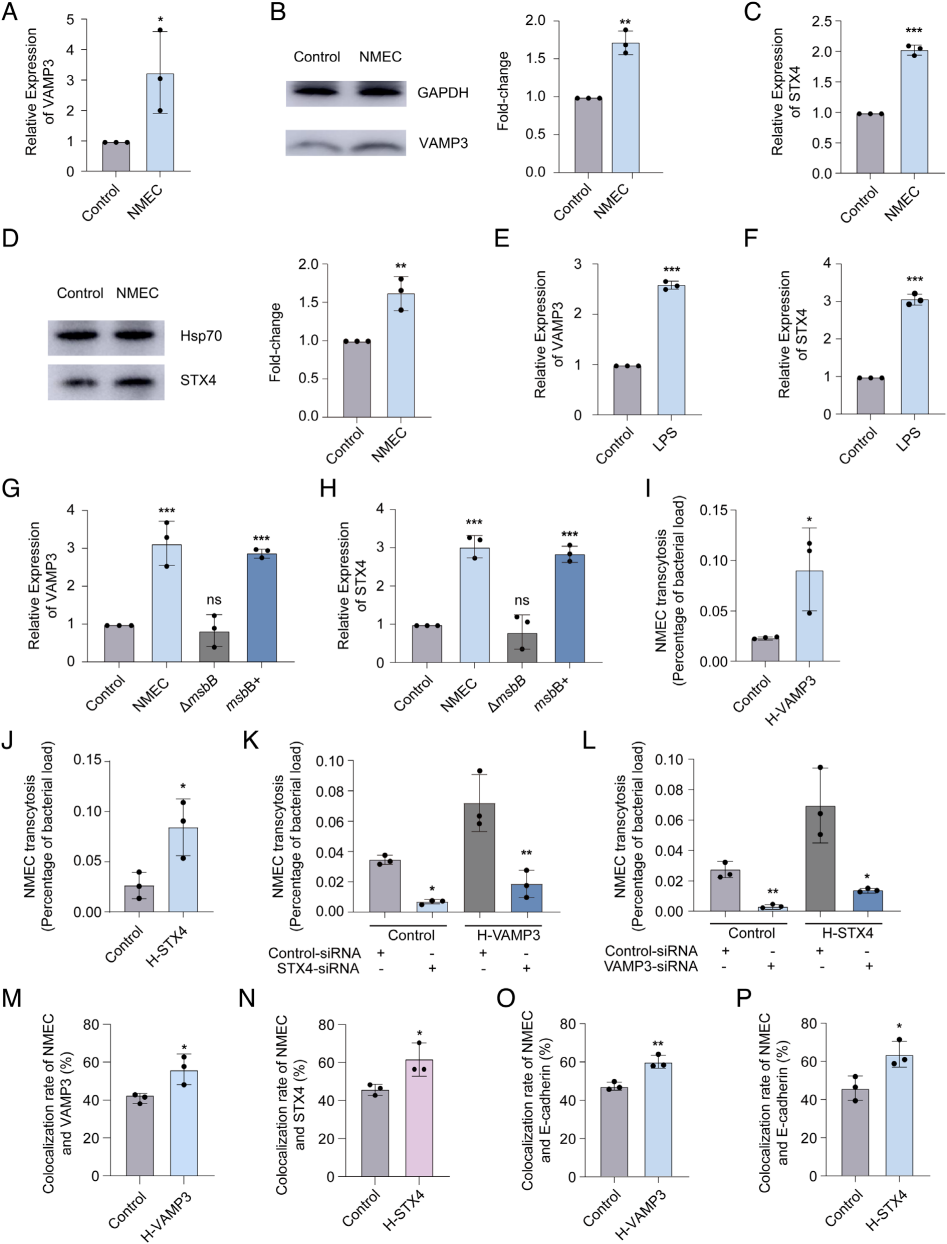
NMEC increases its transcytosis efficiency by enhancing the expression of VAMP3 and syntaxin 4 (STX4). (*A*) Analysis of VAMP3 expression in HBMECs and HBMECs infected by NMEC using qRT-PCR. (*B*) Representative western blotting image and quantitative analysis of VAMP3 in HBMECs and HBMECs infected by NMEC. GAPDH, loading control. (*C*) Analysis of STX4 expression in HBMECs and HBMECs infected by NMEC using qRT-PCR. (*D*) Representative western blotting image and quantitative analysis of STX4 in HBMECs and HBMECs infected by NMEC. Hsp70, loading control. (*E* and *F*) Analysis of VAMP3 (*E*) or STX4 (*F*) expression in HBMECs and HBMECs treated by LPS using qRT-PCR. (*G* and *H*) Analysis of VAMP3 or STX4 expression in HBMECs and HBMECs infected by NMEC WT, Δ*msbB,* or complemented strain Δ*msbB*+ using qRT-PCR. (*I* and *J*) Transcytosis of NMEC across H-VAMP3 (*I*) or H-STX4 (*J*) cells compared with control cells. (*K* and *L*) Transcytosis of NMEC across control cells compared with H-VAMP3 cells (K) or H-STX4 cells (*L*) transfected with control siRNA and STX4 siRNA (*K*), or VAMP3 siRNA (*L*). (*M*) Colocalization of intracellular NMEC with VAMP3 in H-VAMP3 cells and control cells. (*N*) Colocalization of intracellular NMEC with STX4 in H-STX4 cells and control cells. (*O* and *P*) Colocalization of intracellular NMEC with E-cadherin in H-VAMP3 (*O*) and H-STX4 (*P*) cells compared with control cells. n = 3 independent experiments. Data are shown as the mean ± SD. *P < 0.05, **P < 0.01, ***P < 0.001, ns, nonsignificant. One-way ANOVA (*G*, *H*, *K*, and *L*) and two-tailed unpaired Student’s *t* test (*A*–*F*, *I*, *J*, and *M*–*P*).

It is known that the expression of VAMP3 and syntaxin 4 is both induced in response to LPS in macrophages (25, 27). We hypothesized that infection of HBMECs by NMEC can induce the expression of VAMP3 and syntaxin 4 via LPS. To test this hypothesis, we analyzed the expression of VAMP3 and syntaxin 4 in response to NMEC infection. qRT-PCR and western blotting assays showed that the expression of VAMP3 and syntaxin 4 is upregulated in NMEC-infected HBMECs (Fig. 5 *A*–*D*). However, as controls, the expression of VAMP8 and syntaxin 5, both of which are also present on the BCVs in HBMECs (14), as well as syntaxin 3, exhibited no significant change in response to NMEC infection (*SI Appendix*, Fig. S5 *A*–*F*). Additionally, we showed that treatment of HBMECs by LPS results in the up-regulation of VAMP3 and syntaxin 4 (Fig. 5 *E* and *F*). In contrast, infection of HBMECs with the NMEC mutant Δ*msbB*, which lacks LPS, did not induce the expression of VAMP3 and syntaxin 4 (Fig. 5 *G* and *H*). Complementation of Δ*msbB* by wild-type *msbB* restored the ability of bacteria to induce VAMP3 and syntaxin 4 expression (Fig. 5 *G* and *H*). These data indicate that NMEC infection induces the expression of VAMP3 and syntaxin 4 via LPS.

Next, we investigated the effect of increased expression of VAMP3 and syntaxin 4 on NMEC transcytosis. We showed that the transcytosis of NMEC through HBMECs stably overexpressing VAMP3 or syntaxin 4 was significantly enhanced compared with control cells (Fig. 5 *I* and *J*). However, silencing VAMP3 or syntaxin 4 inhibited the influence of overexpressing syntaxin 4 or VAMP3 on NMEC transcytosis (Fig. 5 *K* and *L*). It is consistent with that VAMP3 and syntaxin 4 function collaboratively to mediate the transcytosis of NMEC. As a control, stable overexpression of syntaxin 3 in HBMECs had no effect on NMEC transcytosis (*SI Appendix*, Fig. S5*G*). These data indicate that enhanced expression of VAMP3 and syntaxin 4 in response to NMEC infection promotes bacterial transcytosis.

Furthermore, we tried to investigate how the overexpression of VAMP3 and syntaxin 4 increase the transcytosis of NMEC. Confocal microscopy showed that the percent of BCVs colocalized with VAMP3 or syntaxin 4 were increased in HBMECs that stably overexpress VAMP3 or syntaxin 4 compared with control cells (Fig. 5 *M* and *N*). We also found that the colocalization of BCVs with the basolateral membrane was increased in HBMECs overexpressing VAMP3 or syntaxin 4 (Figs. 5 *O* and *P*). As a control, stable overexpression of syntaxin 3 in HBMECs had no effect on the colocalization of BCVs with the basolateral membrane (*SI Appendix*, Fig. S5*H*). These data indicate that the overexpression of VAMP3 or syntaxin 4 increases the proportion of BCVs positive for VAMP3 or syntaxin 4, and this benefits the fusion of BCVs with the basolateral membrane of HBMECs, leading to the enhanced transcytosis of NMEC.

### The Expression of VAMP3 and Syntaxin 4 Expression Is Induced Via the TLR4-TRIF-Dependent Signaling Pathway.

**Fig. 6.**
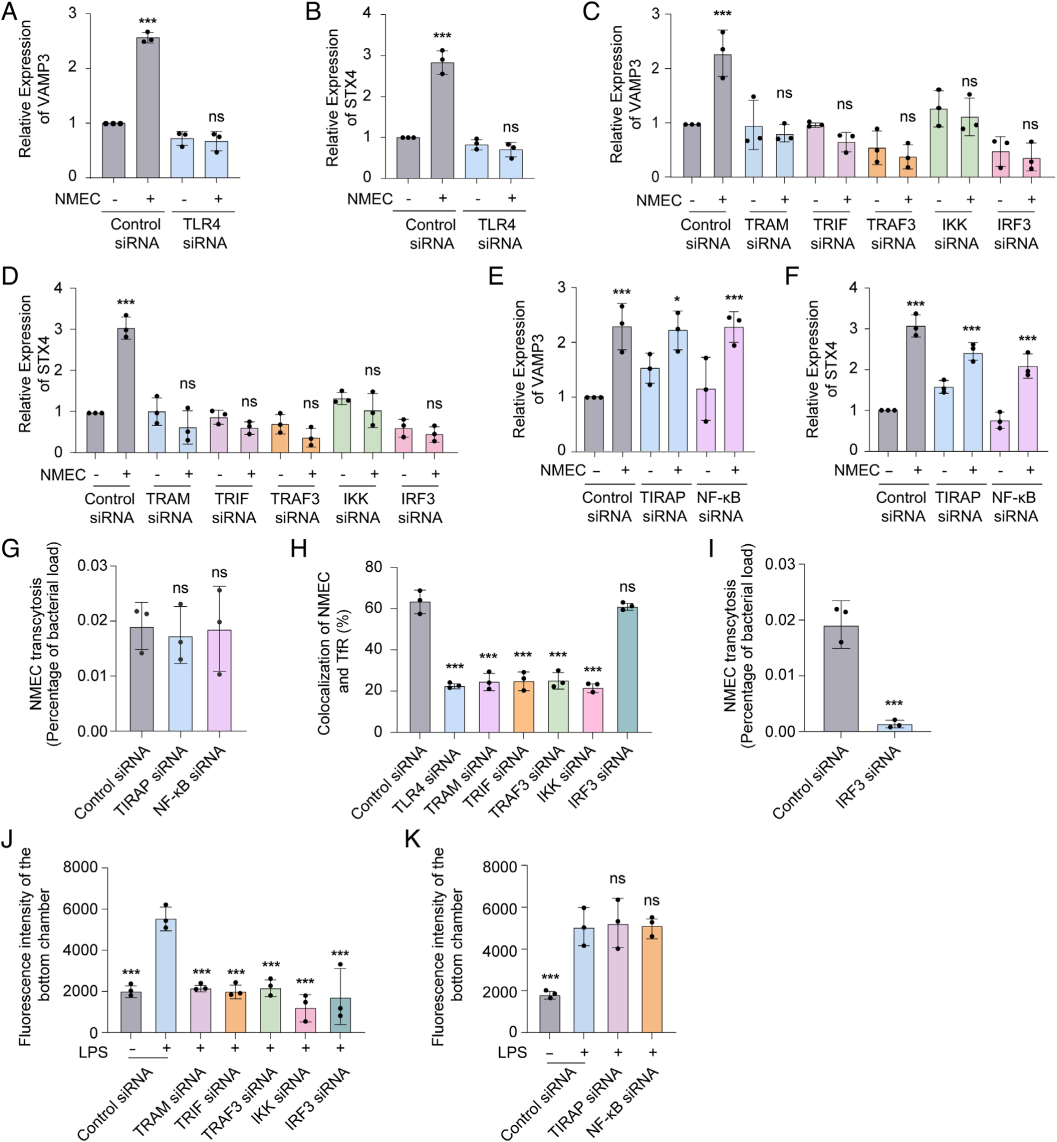
The expression of VAMP3 and syntaxin 4 (STX4) is induced via the TLR4-TRIF-dependent signaling pathway. (*A*–*F*) Analysis of VAMP3 (*A*, *C*, and *E*) and STX4 (*B*, *D*, and *F*) expression in HBMECs transfected with control siRNA or siRNA targeting TLR4 (*A* and *B*), TRAM, TRIF, TRAF3, IKK, IRF3 (*C* and *D*), TIRAP, NF-κB (*E* and *F*) in response to NMEC infection using qRT-PCR. (*G*) Transcytosis of NMEC across HBMECs transfected with control siRNA or siRNA targeting TIRAP or NFκB. (*H*) Colocalization of intracellular NMEC with TfR in HBMECs transfected with control siRNA or siRNA targeting TLR4, TRAM, TRIF, TRAF3, IKK, and IRF3. (*I*) Transcytosis of NMEC across HBMECs transfected with control siRNA or IRF3 siRNA. (*J* and *K*) Spectrophotometric measurement of fluorescence intensity to assess FITC-Tf transcytosis from the apical side to the basolateral side of the HBMECs transfected with control siRNA or siRNA targeting TRAM, TRIF, TRAF3, IKK, IRF3 (*J*), TIRAP or NFκB (*K*) in response to LPS treatment. n = 3 independent experiments. Data are presented as the means ± SD. *P < 0.05; **P < 0.01; ***P < 0.001; ns represents no significance. Two-way ANOVA (*A*–*F*), one-way ANOVA (*G*, *H*, *J*, and *K*), and two-tailed unpaired Student’s *t* test (*I*).

Although it is known that LPS induces the expression of VAMP3 and syntaxin 4 in macrophages, the underlying mechanism remains unclear (25, 27). LPS of Gram-negative bacteria can activate TLR4 signaling of host cells. Consistently, we showed that the upregulation of VAMP3 and syntaxin 4 expression in response to NMEC infection was inhibited when TLR4 in HBMECs was silenced (reduction by 97%, *SI Appendix*, Fig. S6*A*) (Fig. 6 *A* and *B*). TLR4 activates two distinct signaling pathways: the TIRAP-dependent pathway (TLR4-TIRAP-NFκB) and the TRIF-dependent pathway (TLR4-TRAM-TRIF-TRAF3-IKK-IRF3) (28). Next, we investigated which pathway is activated by LPS and TLR4 to induce the expression of VAMP3 and syntaxin 4 in HBMECs. We showed that the upregulation of VAMP3 and syntaxin 4 expression in response to NMEC infection was inhibited when TRAM, TRIF, TRAF3, IKK, or IRF3 in HBMECs was silenced (reduction by 65-98%, *SI Appendix*, Fig. S6*A*) (Fig. 6 *C* and *D*), but not influenced when TIRAP or NFκB was silenced (reduction by 95 to 97%, *SI Appendix*, Fig. S6*A*) (Fig. 6 *E* and *F*). As a control, we showed that the upregulation of *IL1B* expression in response to NMEC was inhibited when TIRAP or NFκB was silenced (*SI Appendix*, Fig. S6*B*), consistent with previous studies (29). It indicates that VAMP3 and syntaxin 4 expression in HBMECs is induced by LPS via the TLR4-TRIF-dependent pathway, but not the TLR4-TIRAP-dependent pathway.

Next, we showed that the transcytosis of NMEC across HBMECs was not influenced when factors involved in the TIRAP-dependent pathway (TIRAP and NFκB) were silenced (Fig. 6*G*). It is consistent with that TIRAP-dependent pathway does not contribute to the induction of VAMP3 and syntaxin 4 expression in response to NMEC infection.

Our previous result showed that several factors within the TRIF-dependent pathway, including TLR4, TRAM, TRIF, and TRAF3, also mediate the fusion of BCV and TfR vesicles in HBMECs (14). It indicates that they may contribute to the transcytosis of NMEC by influencing the fusion of BCV and TfR vesicles. Different from these factors, we showed that silence of IRF3, which is also involved in the TRIF-dependent pathway, did not influence the fusion of BCV and TfR vesicles (Fig. 6*H*). Then, we investigated whether IRF3 influences the transcytosis of NMEC across HBMECs. The results showed that when IRF3 was silenced, the transcytosis of bacteria was significantly inhibited (Fig. 6*I*). It indicates that the TRIF-dependent pathway promotes the transcytosis of NMEC by regulating the expression of VAMP3 and syntaxin 4.

Furthermore, we showed that treatment of HBMECs by LPS, which leads to the increased expression of VAMP3 and syntaxin 4, promoted the transcytosis of Tf (Fig. 5 *E* and *F*), and this effect was inhibited when the TLR4-TRIF-dependent pathway was blocked, but not influenced when the TIRAP-dependent pathway was blocked (Fig. 6 *J* and *K*). It is consistent with that VAMP3 and syntaxin 4 expression is induced in response to LPS via the TLR4-TRIF-dependent pathway, but not the TLR4-TIRAP-dependent pathway.

### IRF3 in the TLR4-TRIF-Dependent Pathway Directly and Indirectly Regulates the Expression of VAMP3 and Syntaxin 4, Respectively.

IRF3 is a regulator absolutely required for the induction of IFN-β and certain IFN-α species (30, 31). It also regulates other inflammatory mediators such as the chemokines CXCL10 and RANTES (32, 33). Therefore, we tried to investigate whether IRF3 directly regulates the expression of VAMP3 and syntaxin 4. Surface plasmon resonance assays using Biacore X100 SPR showed that 6×His-tagged IRF3 interacts with the promoter region of VAMP3, but not the syntaxin 4 promoter (*SI Appendix*, Fig. S7 *A*–*C*). Using a dye-based DNase I foot-printing assay, we revealed a specific IRF3-bound sequence containing a 12-base-pair motif (5′-AAATGGACTTCC-3′) in the promoter region of VAMP3 (*SI Appendix*, Fig. S7*D*), which is located −465bp to −454bp from the proximal transcriptional start site and exhibits similarity to the reported motif bound by IRF3(34). We also performed ChIP-qPCR assays to analyze the enrichment of the VAMP3 promoter in IRF3-ChIP samples compared with mock-ChIP control. The results showed that the VAMP3 promoter was significantly enriched in DNA binding to IRF3 compared to control DNA (*SI Appendix*, Fig. S7*E*). These data indicate that IRF3 directly regulates the expression of VAMP3 by binding to its promoter. However, the regulation of syntaxin 4 expression by IRF3 is indirect, and its underlying mechanism could be a subject of future research.

## Discussion

TfR transcytosis across the BBB has been regarded as one of the most promising routes for drug delivery to the brain and is exploited by dominant meningitis-causing bacterial pathogens for brain penetration. Understanding the mechanisms that determine the efficiency of this process has become one of the most critical issues in improving brain drug delivery systems and can provide insights for developing therapies against bacterial meningitis. In this study, we revealed a crucial step determining the efficiency of TfR transcytosis across the BBB: The interaction between VAMP3 and syntaxin 4 mediates the fusion of TfR vesicles with the basolateral membrane of HBMECs, resulting in the release of Tf from the vesicle into the brain side. Moreover, this mechanism also contributes to the penetration of NMEC into the brain, and NMEC infection induces the expression of VAMP3 and syntaxin 4 via LPS-stimulated TLR4 signaling. Crucially, we provided experimental evidence that overexpression or silence of VAMP3 and syntaxin 4 enhances or reduces the transcytosis of Tf and NMEC across HBMECs. These findings not only provide valuable insights for improving CNS drug delivery system based on TfR transcytosis but also offer clues for establishing effective strategies for the treatment of NMEC infection.

Holo-Tf enters HBMEC through clathrin-mediated endocytosis, forming TfR vesicles carrying holo-Tf. These endocytosed TfR vesicles are sorted into two distinct pathways: recycling or transcytosis(6). Approximately 90% of the endocytosed TfR vesicles are sorted for recycling. The iron is released from Tf of these vesicles and subsequently transported across the basal membrane of HBMECs by ferroportin (15, 35), which is the major pathway for iron transport across the BBB. The resulting apo-Tf–TfR vesicles are retro-transcytosed to the apical surface of HBMECs (6). Besides this pathway, a minor fraction of endocytosed holo-Tf–TfR vesicles are sorted for transcytosis, which traffic to the basolateral membrane of HBMECs along intracellular tubules (36). Subsequently, the new TfR vesicles carrying holo-Tf that emerge from the tubules fuse with the basolateral membrane of HBMECs, resulting in the release of iron into the brain. Overexpression of Rab17 is able to promote TfR transcytosis by enhancing tubule formation (36). Additionally, the membrane-sculpting protein syndapin-2 promotes the transcytosis across the BBB by stabilizing intracellular tubules (37). In this study, we demonstrated that VAMP3 on TfR vesicles determines the final membrane fusion step of TfR transcytosis across HBMECs. However, it remains unknown when VAMP3 is recruited to TfR vesicles—whether it is recruited soon after the entrance of TfR vesicles into HBMECs or after the emergence of new TfR vesicles from intracellular tubules. Furthermore, we showed that overexpression of VAMP3 can increase the fusion between TfR vesicles and the basolateral membrane, by increasing the proportion of TfR vesicles carrying VAMP3 within HBMECs. This resulted in a significant increase of TfR transcytosis efficiency across HBMECs. However, when analyzing the colocalization of VAMP3 with TfR, we did not differentiate TfR vesicles sorted for recycling or transcytosis. It remains unclear whether VAMP3 is specifically recruited to TfR vesicles sorted for transcytosis. These unresolved issues require further investigation in future studies.

A major challenge to the development of biologics-based therapeutics for treating brain disorders is the inability of large molecules to effectively cross the BBB (7, 8). TfR transcytosis has proven to be a vital strategy for delivery of therapeutics, such as the conjugates of antibodies targeting TfR and drugs, across the BBB, which have shown promising results in clinical trials (8, 10, 11). A major challenge in this drug delivery approach lies in the relatively low efficiency of TfR transcytosis, despite numerous efforts to improve the efficiency of this delivery system using antibody engineering technology (10, 12, 13). Our work showed that overexpression of VAMP3 and syntaxin 4 increased the transport of Tf across HBMECs. This indicates that enhancing the expression of VAMP3 and syntaxin 4 in the BBB, employing techniques such as the adeno-associated virus-based technology, has the potential to increase the efficiency of the TfR transport system for delivering drugs to the brain. Furthermore, we demonstrated that the regulator IRF3 directly or indirectly induces the expression of VAMP3 or syntaxin 4, and a specific IRF3 binding site on the VAMP3 promoter was also identified by us. These findings offer potential targets for modulating the expression of VAMP3 and syntaxin 4, thereby enhancing the efficiency of the TfR transcytosis-mediated drug delivery system.

Bacterial meningitis remains a significant cause of mortality and morbidity in neonates worldwide. Despite advances in neonatal care, neonatal meningitis still leads to a mortality rate of 10 to 15% and neurological sequelae in 30 to 50% of cases (38, 39). The emergence and spread of multidrug-resistant strains have diminished the efficacy of antibiotic treatment for neonatal bacterial meningitis. NMEC stands as one of the top two causes of neonatal meningitis (40–43). Traversal of the HBMECs is the key step in the pathogenesis of bacterial meningitis (44). Recently, we found that NMEC couple its penetration of BBB to TfR transcytosis by activating the fusion process of BCVs with TfR vesicles within HBMECs (14). However, as a major portion of endocytosed TfR vesicles are shuttled back to the apical membrane of HBMECs, most invaded NMEC is exocytosed back to the extracellular milieu by HBMECs (14), which is similar to the protective cell-autonomous immune response utilized by other nonimmune cells to clear invaded pathogens (45). Consequently, only a small portion of invaded NMEC is able to penetrate the BBB. In this study, we found that VAMP3 at TfR-NMEC vesicles and syntaxin 4 that is limited to the basolateral membrane of HBMECs contributes to bacterial transcytosis by mediating the fusion of TfR-NMEC vesicles with the basolateral membrane. We further found that NMEC cleverly enhances its transcytosis efficiency across the BBB, through inducing VAMP3 and syntaxin 4 expression via the TLR4-TRIF-dependent pathway. Consistently, we showed that silencing VAMP3 and syntaxin 4 significantly reduces the transcytosis of NMEC in the human BBB model in vitro. Moreover, penetration of NMEC into the brain was significantly inhibited in VAMP3-deficient mice. These findings could provide insights for developing effective strategies to combat NMEC infection.

VAMP3-deficient mice have been reported to exhibit a relatively mild phenotype, which is likely due to compensation by other SNARE proteins, such as VAMP2 and VAMP8 (46). However, it is important to note that in certain organs or cell types, the deficiency of VAMP3 cannot be fully compensated by other SNARE proteins (47, 48). For instance, the knockout of VAMP3 in mice affects the trafficking of NKCC2 (Na^+^/K^+^/2Cl^-^ cotransporter) in renal cells of the thick ascending limb. This effect is not compensated by other SNARE proteins (47). We showed that silencing or overexpressing VAMP2 or VMAP8 in HBMECs did not alter the transcytosis of Tf, nor did it influence the colocalization of TfR with the basolateral membrane or syntaxin 4 in polarized HBMECs (Figs. 1*A* and *SI Appendix*, Fig. S8 *A*–*E*). Additionally, silencing or overexpressing VAMP2 or VMAP8 also had no effect on NMEC transcytosis or on the colocalization of BCVs with the basolateral membrane of HBMECs (*SI Appendix*, Fig. S8 *F*–*I*). Furthermore, we showed that silencing VAMP2, VAMP8, or syntaxin 3 had no effect on the colocalization of TfR or BCVs with VAMP3 (*SI Appendix*, Fig. S8 *J* and *K*). Collectively, these data indicate that VAMP2 and VAMP8 do not contribute to TfR transcytosis through HBMECs, and cannot compensate for VAMP3 in this context. This may be related to the specific features of polarized HBMECs, which could be the subject of future studies. In addition, it is worth noting that, using inductively coupled plasma mass spectroscopy, we found that iron levels in the brains of VAMP3-deficient mice were not significantly different from those in wild-type mice (*SI Appendix*, Fig. S8*L*). This is consistent with the notion that ferroportin-mediated export of iron is the primary pathway for iron transport across the BBB (15, 35).

The special localization of the t-SNARE syntaxin 4 at the basolateral membrane of HBMECs directly determines the unidirectional transport of holo-Tf from the circulating blood into the brain. Syntaxin 4 is found on the plasma membrane of a number of cell types and is involved in the fusion of incoming vesicles or secretory granules at the cell surface as the final step in secreting cytokines and other cargo (49, 50). The localization of syntaxin 4 in polarized cells is seldom reported, with the exception that it is identified to be localized at the basolateral membrane of MDCK cells (24). In this study, using confocal microscopy analysis, we first revealed that syntaxin 4 is limited to the basolateral membrane in HBMECs, where it interacts with VAMP3 present on TfR vesicles and TfR-NMEC vesicles to mediate the transcytosis of Tf and NMEC. In other cells, syntaxin 4 also interacts with other v-SNAREs, such as VAMP2, VAMP7, and VAMP8, to regulate membrane fusion events (51–53). It is likely that syntaxin 4 at the basolateral membrane of HBMECs also plays a role in the transcytosis of other molecules across HBMECs, which could be a topic for future research.

Our recent work demonstrated that SNAP23 on TfR vesicles initiates the fusion of NMEC-containing vesicles and TfR vesicles within HBMECs, coupling bacterial penetration of the BBB to TfR transcytosis(54). In this study, we showed that SNAP23 is probably involved in the final membrane fusion step of TfR transcytosis across the BBB by interacting with VAMP3 and syntaxin 4 (*SI Appendix*, Fig. S2 *H* and *I*). Thus, it is likely that SNAP23 facilitates NMEC penetration of the BBB by mediating two distinct steps: the fusion between TfR vesicles and BCVs and the fusion of TfR-NMEC-vesicles with the basolateral membrane of HBMECs.

TLR4 signaling is known to regulate the formation of SNARE complexes at the posttranslational level (55, 56). For example, TLR4 signaling promotes the phosphorylation of phagosome-associated SNAP23 in dendritic cells, which stabilizes SNARE complexes and thereby facilitates the delivery of major histocompatibility complex class I from recycling endosomes to phagosomes (57). In this study, we showed that SNAP23 interacts with VAMP3 and syntaxin 4, and also contributes to TfR transcytosis. It is likely that, in addition to inducing the expression of VAMP3 and syntaxin 4, TLR4 signaling also triggers the phosphorylation of SNAP23 in HBMECs, which may enhance TfR transcytosis by promoting the formation of VAMP3-syntaxin 4-SNAP23 complexes. Furthermore, phosphorylation of other v-SNAREs and t-SNAREs has also been observed (56). It is possible that TLR4 signaling influences the interaction between VAMP3 on TfR vesicles and syntaxin 4 at the basolateral membrane of HBMECs through regulating the phosphorylation status of these SNARE proteins. These possibilities warrant further investigation in future studies.

## Materials and Methods

All animal experiments were performed according to the standards set forth by the Guide for the Care and Use of Laboratory Animals. All animal studies were conducted according to protocols approved by the Institutional Animal Care Committee of Nankai University (Tianjin, China) and performed under protocol no. IACUC 2016030502. Details of primers, siRNAs, cell lines, bacterial strains, and experimental methods, including cell culture and transfection, NMEC invasion assays, Transwell assay, immunofluorescence microscopy, penetration of Tf–biotin across the BBB of mice, in vivo bacterial penetration of the BBB, western blotting analysis, coimmunoprecipitation analysis, quantitative RT-PCR, Dye primer-based DNase I footprinting assay, ChIP-qPCR, surface plasmon resonance assay, quantification, and statistical analysis are described in *SI Appendix*, Tables S1 and *Materials and Methods*.

## Supplementary Material

Appendix 01 (PDF)

## Data Availability

All study data are included in the article and/or *SI Appendix*.
