# Supplementary material for: Vamp3/syntaxin 4 mediates the basolateral membrane fusion of TfR transcytosis across the BBB and is exploited by pathogenic Escherichia coli
Source: Proc Natl Acad Sci U S A. 2025 Jul 2;122(27):e2500285122. doi: 10.1073/pnas.2500285122 (PMC12260585; doi:10.1073/pnas.2500285122)
Supplement: Supplementary file 1 — Appendix 01 (PDF) [file pnas.2500285122.sapp.pdf]

PNAS

Supporting Information for

Vamp3/syntaxin 4 mediates the basolateral membrane fusion of TfR transcytosis across the BBB and is exploited by pathogenic *E. coli*

Bin Liu[a,b,c,1], Yingying Su[a,b,1], Hao Sun[a,b,1], Bin Yang[a,b,1], Lili Wan[d], Xiaoya Li[a,b], Shaobin Hou[d], Guozhen Ma[a,b], Juan Joanna Yu[d], Lu Feng[a,b,c,2], Huamin Henry Li[d,2], Lei Wang[a,b,e,2]

[a] National Key Laboratory of Intelligent Tracking and Forecasting for Infectious Diseases, TEDA Institute of Biological Sciences and Biotechnology, Nankai University, Tianjin 300457, China

[b] The Key Laboratory of Molecular Microbiology and Technology, Ministry of Education, Nankai University, Tianjin 300071, China

[c] Nankai International Advanced Research Institute, Nankai University Shenzhen, Shenzhen 518045, China

[d] Institute for Asthma and Allergy, Chevy Chase, MD 20815

[e] Southwest United Graduate School, Kunming 650092, China

[1] These authors contributed equally: Bin Liu, Yingying Su, Hao Sun and Bin Yang

[2] To whom correspondence may be addressed. E-mail: fenglu63@nankai.edu.cn, henry@allergyasthma.us or wanglei@nankai.edu.cn.

This PDF file includes:

Supporting text

Figures S1 to S9

Tables S1

SI References

Materials and Methods

Cell culture conditions and transfections

The HBMEC cell line (Innoprot, Bizkaia, Spain; #P10361-IM), which is a fully de-identified cell line that does not contain any personally identifiable information, was cultured in Dulbecco's Modified Eagle Medium (DMEM)(Gibco) supplemented with 10% fetal bovine serum (FBS)(Excell Bio), 10% Nu-Serum (BD Biosciences), 100 units/mL penicillin, 100 μg/mL streptomycin and incubated at 37°C in 5% CO_2. To stably knock-down gene expression, shRNA targeting the specific gene or a scrambled control shRNA was designed and constructed into the lentiviral vector pGMLV-SC5 (Genomeditech). 293T cells were used for lentivirus packaging with HBMECs. To stably overexpress gene expression, the indicated genes were cloned into the vector lenti-CMV-MCS-PGK-Puro (Genomeditech). For silencing gene expression transiently, HBMECs were transfected with negative control siRNA or siRNA targeting specific genes using Lipofectamine RNAi MAX (Invitrogen) according to the manufacture instruction.

Bacterial strains

E. coli strain RS218, a prototype meningitis isolate from the CSF of a newborn infant, was designated as NMEC (1). Mutant Δ*msbB* was generated from NMEC using the λ-Red recombination system (2). The complementary strain Δ*msbB*+ was established

by cloning *msbB* and native promoters into the pACYC-184 plasmid, which was then transformed into NMEC.

Animal model

The experimental protocols were approved by the Institutional Animal Care Committee at Nankai University. 18-day-old C57BL/6 mice were purchased from Beijing Vital River Laboratory Animal Technology Co., Ltd. Gene-deficient VAMP3$^{-/-}$ mice were obtained from Model Organisms Center (Shanghai, China) and maintained on a C57BL/6J background.

NMEC invasion assays

Bacterial strains were grown overnight in LB broth at 37°C and activated to the exponential phase at an optical density of 600 nm ($OD_{600\ nm}$) of 0.6. Bacteria were pelleted, washed with PBS and resuspended in infection medium [medium 199 (Gibco) and Ham's F-12 (Gibco) (1:1) supplemented with 5% FBS]. HBMECs were infected with bacteria at a multiplicity of infection (MOI) of 100:1 for 90 min, followed by washing three times with PBS. Cells were then incubated in DMEM medium containing 100 μg/mL gentamycin for 60 min to kill extracellular bacteria. Then, the cells were washed with sterile PBS three times, lysed using 0.1% Triton X-100 in PBS, and the bacterial load was enumerated by LB agar plates. Invasion efficiency was calculated as the percentage relative to WT HBMECs, which was set at 100%. All assays were performed with at least three independent biological replicates.

Transwell assay

HBMECs were cultured on the inner surface of collagen-coated Transwell inserts (pore size 3.0 μm), containing 200 μL DMEM medium with 10% FBS and 10% Nu-Serum, and 1 mL DMEM medium with 10% FBS and 10% Nu-Serum was added to the lower chamber. HBMECs were seeded at a density of 2×10^5 cells/well in 200 μL medium onto 24-well Transwell chambers (Corning-Costar). Then, HBMECs were cultured to confluence for at least 5 days. The integrity of the monolayers was monitored by measuring transendothelial electrical resistance (TEER) using an ECIS TEER24 machine (Applied Biophysics Inc). No significant changes in TEER were observed under experimental conditions, indicating that the polarized monolayers in the transwells were intact.

For FITC-Tf permeability assays, 0.1 mg/ml FITC-Tf (Thermo Fisher Scientific) was added to the upper chamber, while fresh serum-free media were added to the lower compartment. After a 90-minute incubation, both the apical and basolateral chambers were washed three times with sterile PBS and replenished with DMEM medium, followed by an additional 6-hour incubation at 37°C. Fluorescence in the lower compartment was measured at excitation 495 nm and emission 528 nm using a SpectraMax M5 microplate reader (Molecular Devices). All experiments were performed in triplicate.

For NMEC transcytosis, bacteria in 200 μL infection medium were added to the endothelial cell layers on Transwell filters at a MOI of 100:1 and incubated for 90

min. Both apical and basolateral chambers were washed with sterile PBS three times and extracellular bacteria were killed by incubation in DMEM medium containing 100 μg/mL gentamycin for 60 min. After washing by sterile PBS three times, both apical and basolateral chambers were replenished with DMEM medium containing 25 μg/mL trimethoprim and 50 μM rottlerin to prevent bacterial reattachment or reentry. After incubation for additional 4 hours at 37°C, 100 μL samples were collected from the apical and basolateral chambers, respectively, and plated on agar for enumeration. In addition, HBMECs on the Transwell filter were lysed with 0.1% Triton X-100, and samples were also collected and enumerated by plating suitable dilutions on agar plates. The percentage of bacterial transcytosis was calculated as the proportion of bacteria in the basolateral chamber relative to the total bacterial count (apical, basolateral, and filter). All experiments were performed in triplicate.

Immunofluorescence microscopy

HBMECs were seeded on 20-mm diameter coverslips and cultured in DMEM medium at 37°C. Cells were fixed with ice-cold 4% paraformaldehyde at 4°C for 10 min, then permeabilized with 0.3% Triton for 20 min and blocked with 5% BSA at room temperature for 1 h. Cells were incubated with primary antibodies diluted in 5% BSA at 4°C overnight. To visualize with a confocal microscopy, cells were washed with PBS three times and then incubated with respective fluorophore-conjugated secondary antibodies at room temperature for 1 h. Then the cells were washed three

times with PBS and mounted using Prolong Gold Antifade Mountant with DAPI (Thermo Fisher Scientific).

When bacterial infection is included in the experiments, HBMECs were seeded on coverslips and cultured in DMEM medium at 37°C for 24 h prior to infection. After 90 min incubation with NMEC at a MOI of 100:1, the cells were processed as the above steps, and stained with the appropriate antibodies and imaged by confocal microscopy.

For polarized HMBECs, the cells were grown on collagen filters, fixed in 4% paraformaldehyde at 4°C for 10 min, permeabilized with 0.3% Triton for 20 min, and blocked with 5% BSA at room temperature for 1 h. Cells were stained with primary antibodies diluted in 5% BSA at 4°C overnight and fluorophore conjugated secondary antibodies at room temperature for 1 h. After staining, Transwell membranes containing cells were excised with a scalpel and placed onto glass slides with glass coverslips and Prolong Gold Antifade Mountant with DAPI.

Primary antibody used include: anti-VAMP3 antibody (1:100 dilution, Invitrogen, HB201-6A1), anti-STX4 antibody (1:100 dilution, Abcam, ab246856), anti-TfR (1:100 dilution, Abcam, ab84036), anti-E-cadherin (1:100 dilution, Abcam, ab231303), anti-gp135 (1:100 dilution, Invitrogen, PA5-28116).

Three slides were examined for each sample in the above assay. 10 random areas or 100-150 BCVs were observed on each slide. Images were taken using a Zeiss

LSM800 confocal microscope (Zeiss). The colocalization Mander's and Pearson's coefficients were analysed using ImageJ.

Penetration of Tf-biotin across the BBB of mice

Each mouse received Tf-biotin via tail vein as described previously (3). Four hours post-injection, CSF samples were collected for quantitation of Tf-biotin using a Fluorescence Biotin Quantitation Kit (Thermo Fisher Scientific) according to the manufacture's instruction.

***In vivo* bacterial penetration of the BBB**

C57BL/6 wild type mice and gene-deficient VAMP3$^{-/-}$ mice were used for the tail vein injection model. Mice were injected intravenously with 1×10^7 CFU of NMEC in 100 μL sterile PBS, a dosage sufficient to induce high levels of bacteremia and subsequent bacterial traversal of the BBB, mimicking the pathogenesis of human meningitis (4). At 4 h after bacterial inoculation, blood and CSF samples were collected for bacterial cultures. The bacterial numbers in the samples were determined by plating serial dilutions on LB agar plates.

Western blotting analysis

Proteins were separated by 4–12% SDS-PAGE and transferred onto 0.22 μm PVDF membranes (Millipore). Membranes were blotted with 5% non-fat milk in TBST (m/v) at 25°C for 1 h and incubated overnight at 4°C with the appropriate primary

antibodies. The membranes were washed three times with TBST, then incubated for 1 h at 25°C with HRP-conjugated goat anti-rabbit or -mouse IgG antibodies. Primary antibody used include: anti-VAMP3 antibody (1:1000 dilution, Invitrogen, HB201-6A1), anti-STX4 antibody (1:1000 dilution, Abcam, ab246856), anti-Hsp60 (1:1000 dilution, Abcam, ab59457), anti-GAPDH (1:1000 dilution, Abcam, ab128915), anti-Hsp70 (1:1000 dilution, Abcam, ab2787), anti-VAMP1 antibody (1:1000 dilution, Abcam, ab151712), anti-VAMP2 antibody (1:1000 dilution, Abcam, ab181869), anti-VAMP4 antibody (1:1000 dilution, Abcam, ab290726), anti-VAMP7 antibody (1:1000 dilution, Abcam, ab36195), anti-VAMP8 antibody (1:1000 dilution, Abcam, ab76021), anti-STX5 antibody (1:1000 dilution, Abcam, ab211417), anti-TLR4 antibody (1:1000 dilution, Santa Cruz Biotechnology, sc-293072), anti-TRAM antibody (1:1000 dilution, Abcam, ab190982), anti-TRIF antibody (1:1000 dilution, Abcam, ab13810), anti-TRAF3 antibody (1:1000 dilution, Abcam, ab36988), anti-IKK antibody (1:1000 dilution, Abcam, ab124766), anti-IRF3 antibody (1:1000 dilution, Abcam, ab68481), anti-TIRAP antibody (1:1000 dilution, Abcam, ab17218), anti-NF-κB antibody (1:1000 dilution, Abcam, ab288751), anti-syntaxin 3 (1:1000 dilution, Abcam, ab133750), anti-STX18 (1:1000 dilution, Abcam, ab156017). Chemiluminescent detection was performed using SuperSignal West Pico substrate (Thermo Fisher Scientific). Images were acquired using an Amersham™ Imager 600 System (General Electric Company), and protein levels were quantified using ImageJ software. Original images are provided in Fig. S9.

Co-immunoprecipitation (co-IP) analysis

Proteins were extracted from HBMECs, and immunoprecipitation was performed using 2 μg polyclonal antibody against mouse FLAG (1:1,000, Beyotime Biotechnology). After 3 hours of incubation, protein A magnetic bead (Thermo Fisher Scientific) was added and incubated overnight at 4 °C, followed by centrifugation at 12,000 g for 1 min. The precipitates were rinsed four times with immunoprecipitation buffer (0.5% NP-40, Tris-Cl pH8.0, 0.15 M NaCl) to remove non-specific binding. Protein levels in the precipitates were analyzed by Western blotting.

Quantitative RT-PCR (qRT-PCR)

Total RNA was extracted following standard protocols, and RNA was treated with DNase I at 37°C for 30 min to eliminate DNA contamination. cDNA was synthesized using the PrimeScript™ RT reagent Kit (Takara; RR037A) according to the manufacturer's instructions. GAPDH was used as a reference to standardize expression across the samples. Amplification and detection were performed using SYBR green dye on an Applied Biosystems ABI 7500 sequence detection system (Applied Biosystems). Relative difference in gene expression was calculated using the cycle threshold method ($2^{\Delta\Delta ct}$). Data were collected from at least three biological replicates.

Dye primer-based DNase I footprinting assay

A 1000-bp fragment of the VAMP3 promoter regions was generated by PCR with 6-FAM-labeled primers (*Table S1*). The coding sequence of human IRF3 was cloned

into pET32a plasmid and transformed into *E. coli* BL21 (DE3). The expression of IRF3 with an N-terminal 6× His tag was induced with 0.5 mM IPTG for 6 h at 16°C, and the protein was purified using a HiTrap Ni^{2+} chelating column (GE Healthcare). Protein concentrations were determined by the Bradford protein assay (Bio-Rad). Different amounts of 6×His-tagged IRF3 were added to 40 ng of 6-FAM-labeled VAMP3 promoter in binding buffer (10 mM Tris-HCl [pH 7.5], 0.2 mM dithiothreitol, 5 mM $MgCl_2$, 10 mM KCl, and 10% glycerol). 0.05 U DNase I was added to the 20 μL reaction for 15 min at 30°C. The reaction was stopped by heating at 70°C for 10 min in the presence of 250 mM EDTA. DNA fragments were purified with the QIAquick PCR Purification Kit (Qiagen) and analyzed by MAP Biotech Co., Ltd. using a peak scanner (Applied Biosystems).

ChIP-qPCR

HBMECs were transfected with pcDNA3.1 overexpressing IRF3-FLAG or an empty vector using Lipofectamine 3000 (Invitrogen) according to the manufacture's instruction. Cells were collected at 48 h post transfection, and 1% formaldehyde (v/v) was added immediately to cross-link proteins and DNA. After 25 min of incubation, the cross-linking was quenched with 0.5 M glycine. After washing three times in ice-cold sterile PBS, cells were resuspended in lysis buffer (50 mM Tris-HCl pH 7.5, 100 mM NaCl, 1 mM EDTA, 1 mM PMSF, 20 mg/mL lysozyme). After incubation at 37°C for 30 min, samples were sonicated to generate DNA fragments of approximately 250 to 500 bp. Insoluble cellular debris was removed via centrifugation

for 20 min at 4°C, and the supernatant was collected for immunoprecipitation (IP) experiments as the input sample. The mock and IP samples were incubated with anti-mouse IgG and anti-FLAG antibodies (Sigma), respectively, and then incubated with protein A magnetic beads (Invitrogen). Protein-DNA complexes were washed, reversed and purified with a PCR purification kit (Qiagen). To measure the enrichment of potential IRF3-binding targets in the immunoprecipitated DNA samples, the percent of the input and fold enrichment were determined using SYBR green PCR master mix. Relative target levels were calculated using $2^{-\Delta\Delta Ct}$ method, with *GAPDH* as a negative control. Results represent the average of three independent biological replicates.

Surface plasmon resonance (SPR) assay

SPR measurements were performed using streptavidin-coated sensor chips (SA chip) on a Biacore™ X100 analytical system (GE Healthcare). SA-chip (Biacore™) was activated by injection of a mixture containing 50 mM NaOH and 1 M NaCl. The target nucleic acid VAMP3 or syntaxin 4-promoters were 3'-biotinylated and diluted to 100 μg/mL with deionised water at a flow rate of 10 μL/min 600 s and immobilised on the surface of the SA chip. Proteins were dialyzed overnight against HBS-EP+ buffer, and six different concentrations of each protein were prepared by serial dilution with HBS-T buffer for each set of sensorgrams. The procedures for kinetic analyses were automated to perform repetitive cycles of sample injection and regeneration (5). Proteins were diluted in an isocratic gradient with HBS-EP+ buffer.

The sample was analyzed at a flow rate of 30 μl/min. The time of protein binding was 180 s and the time of dissociation was 300 s. At the end of each cycle, the chip surface was regenerated by injecting 0.5% SDS. The sensorgrams were processed for baseline alignment and reference channel subtraction with the Biacore™ X100 Evaluation Software (GE Healthcare). Kinetic analysis was performed by globally fitting the curves describing a simple 1:1 bimolecular model to the set of five sensorgrams.

Measurement of Brain Iron

The total iron content of the brain was measured by inductively coupled plasma mass spectrometry (ICP-MS) as previously described (6). Samples were digested overnight using a microwave digestion system with nitric acid. Total iron content of each sample was determined by ICP-MS (Thermo Fisher Scientific) against a standard. The iron content was normalized to wet weight and the results were reported as μg/g of wet tissue weight.

Quantification and statistical analysis

Data were presented as the mean ± SD. Statistical significance was assessed using GraphPad Prism 9.3.1(GraphPad Inc), using the two-tailed unpaired Student's *t* test, one-way ANOVA, two-way ANOVA, or Mann–Whitney *U*-test according to the test requirements, as described in the figure legends.

Supplementary Figures

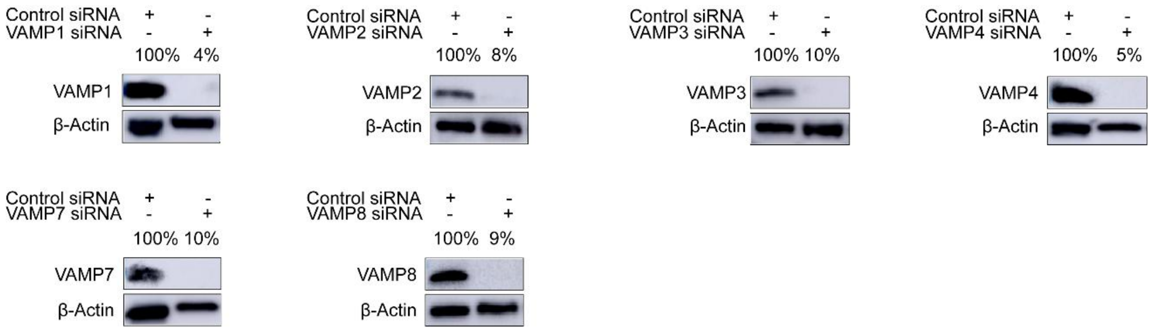

Fig. S1 The effect of silencing VAMP1, VAMP2, VAMP3, VAMP4, VAMP7 and VAMP8 in HBMECs. HBMECs were transfected with siRNA targeting VAMP1, VAMP2, VAMP3, VAMP4, VAMP7, VAMP8, or with control siRNA. The silencing efficiency is analyzed by immunoblotting. β-actin, loading control. n = 3 independent experiments.

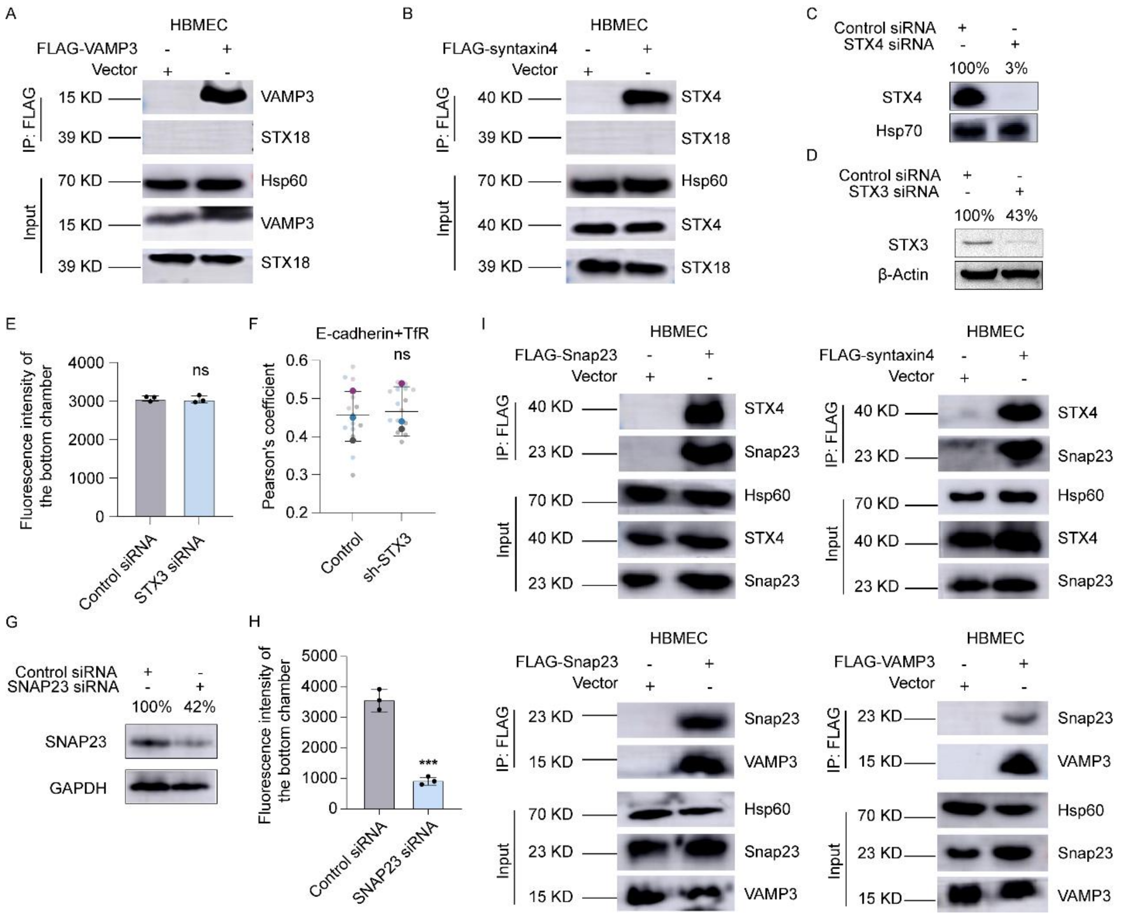

Fig. S2 The interaction of VAMP3 with syntaxin 4 at the basolateral membrane mediates the final fusion step of TfR transcytosis across HBMECs. (A, B) Co-immunoprecipitation assays of syntaxin 18 (STX18) and VAMP3 (A) or STX4 (B) in HBMECs. Hsp60, loading control. n = 3 independent experiments. (C, D) The effect of silencing STX4 or syntaxin 3 (STX3) in HBMECs. HBMECs were transfected with siRNA targeting STX4 (C) or STX3 (D), and control siRNA. The silencing efficiency is analyzed by immunoblotting. Hsp70 or β-actin, loading control. (E) Spectrophotometric measurement of fluorescence intensity to assess FITC-Tf transcytosis from the apical side to the basolateral side of HBMECs transfected with control siRNA or siRNA targeting STX3. (F) Colocalization of E-cadherin with TfR in polarized HBMECs transfected with the pGMLV-SC5 lentiviral vector harboring

shRNA targeting STX3 (sh-STX3) or control shRNA. Quantification of colocalization was shown by calculating the Pearson correlation coefficients. The IF data are quantified using Superplot, which concisely visualizes individual data points and their averages. The distinct combinations of colors indicate the three independent experiments performed. Horizontal lines show mean ± SD. Each small dot in the graph corresponds to a specific data point representing an analyzed image. The larger dots represent the average values calculated from the respective data points. (G) The effect of silencing SNAP23 in HBMECs. HBMECs were transfected with siRNA targeting SNAP23, or with control siRNA. The silencing efficiency is analyzed by immunoblotting. Hsp60, loading control. (H) Spectrophotometric measurement of fluorescence intensity to assess FITC-Tf transcytosis from the apical side to the basolateral side of HBMECs transfected with siRNA targeting SNAP23, or with control siRNA. (I) Co-immunoprecipitation assays of SNAP23 and VAMP3 or STX4 in HBMECs. Hsp60, loading control. n = 3 independent experiments. Data are shown as the mean ± SD. *** $P < 0.001$, ns, nonsignificant. Two-tailed unpaired Student's *t* test (E, F, H).

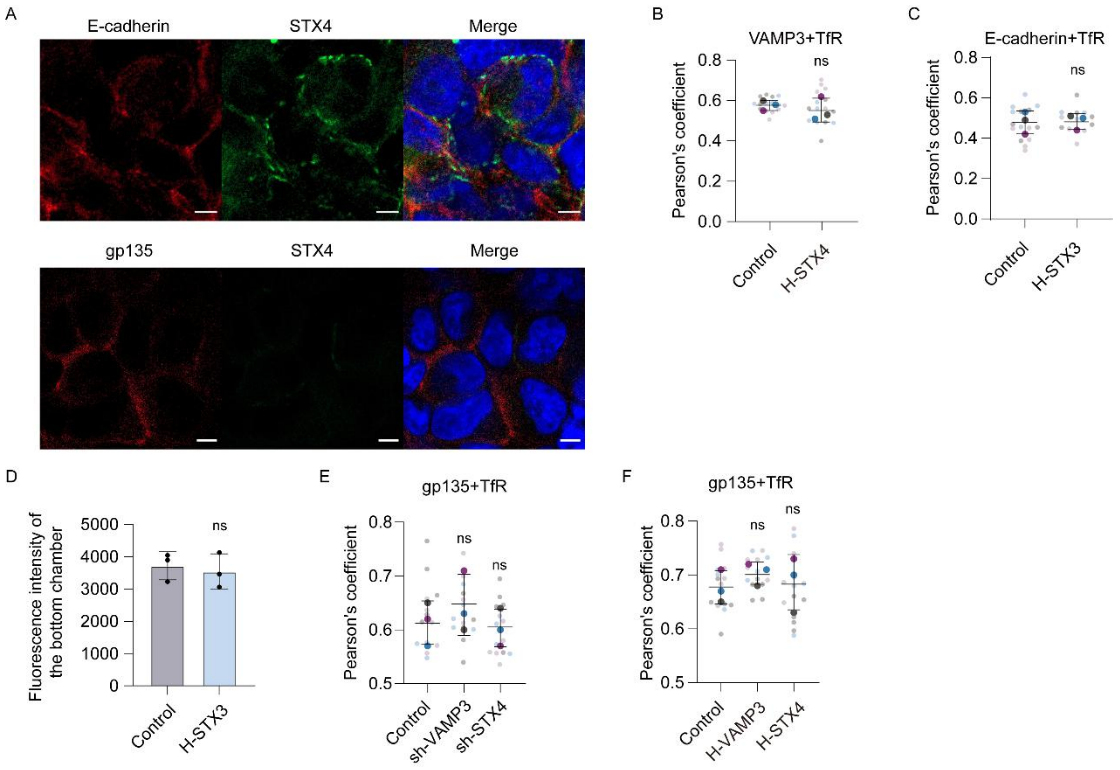

Fig. S3 Overexpression of VAMP3 and STX4 enhances the efficiency of TfR transcytosis. (A) Colocalization of STX4 (green) with E-cadherin (red, top) or gp135 (red, bottom) in polarized STX4-overexpressing (H-STX4) cells, respectively. Scale bar, 5 μm. (B) Colocalization of VAMP3 with TfR in polarized H-STX4 cells and control cells. (C) Colocalization of E-cadherin with TfR in polarized STX3-overexpressing (H-STX3) cells and control cells. Quantification of colocalization was shown by calculating the Pearson correlation coefficients. (D) Spectrophotometric measurement of fluorescence intensity to assess FITC-Tf transcytosis from the apical side to the basolateral side of H-STX3 cells and control cells. n = 3 independent experiments. (E, F) Colocalization of gp135 with TfR in polarized sh-VAMP3, sh-STX4 cells and control cells (E), or H-VAMP3, H-STX4, and control cells (F). Quantification of colocalization was shown by calculating the Pearson correlation coefficients. The IF data are quantified using Superplot (B, C, E,

F), which concisely visualizes individual data points and their averages. The distinct combinations of colors indicate the three independent experiments performed. Horizontal lines show mean ± SD. Each small dot in the graph corresponds to a specific data point representing an analyzed image. The larger dots represent the average values calculated from the respective data points. Data are shown as the mean ± SD. ns, nonsignificant. One-way ANOVA (E, F), Two-tailed unpaired Student's t test (B-D).

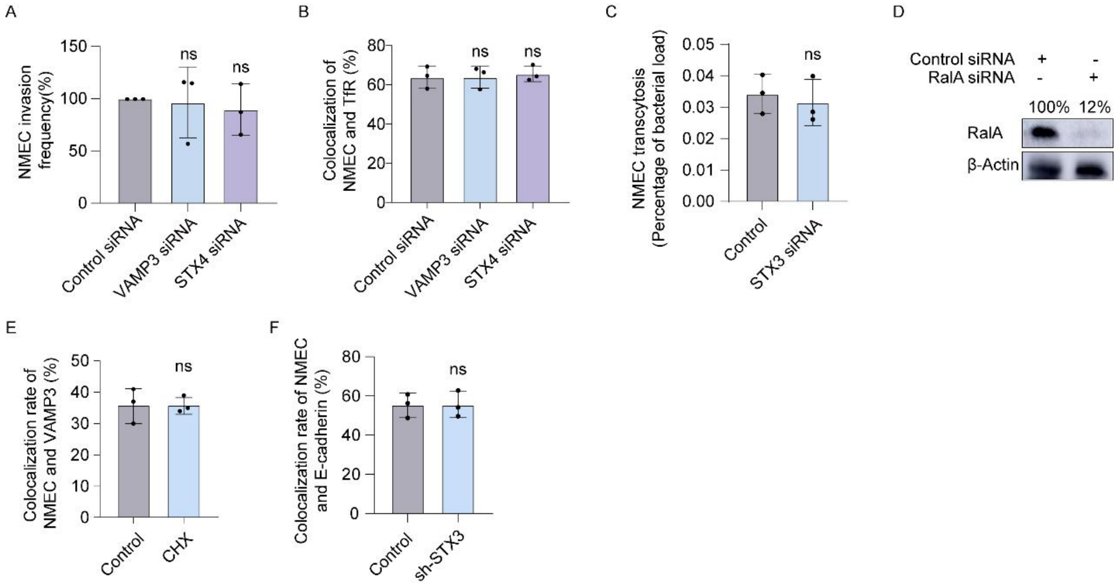

Fig. S4 VAMP3 and STX4 contributes to the transcytosis of NMEC. (A) Bacterial invasion of HBMECs transfected with VAMP3 siRNA, syntaxin 4 (STX4) siRNA or control siRNA. (B) Colocalization of intracellular NMEC with TfR in polarized HBMECs transfected with VAMP3 siRNA, STX4 siRNA or control siRNA. The numbers indicate the percentage of BCVs colocalized with TfR relative to total intracellular BCVs (n = 3 slides). (C) Transcytosis of NMEC across HBMECs transfected with control siRNA or STX3 siRNA. (D) The effect of silencing RalA in HBMECs. HBMECs were transfected with siRNA targeting RalA, or with control siRNA. The silencing efficiency is analyzed by immunoblotting. β-actin, loading control. (E) Colocalization of intracellular NMEC with VAMP3 in HBMECs pretreated with 10 μg/mL cycloheximide. (F) Colocalization of intracellular NMEC with E-cadherin in polarized HBMECs transfected with the pGMLV-SC5 lentiviral vector harboring shRNA targeting STX3 (sh-STX3) or control shRNA. The numbers indicate the percentage of BCVs colocalized with TfR relative to total intracellular BCVs (n = 3 slides). n = 3 independent experiments. Data are shown as the mean ±

SD. ns, nonsignificant. One-way ANOVA (A, B), Two-tailed unpaired Student's *t* test (C, E, F).

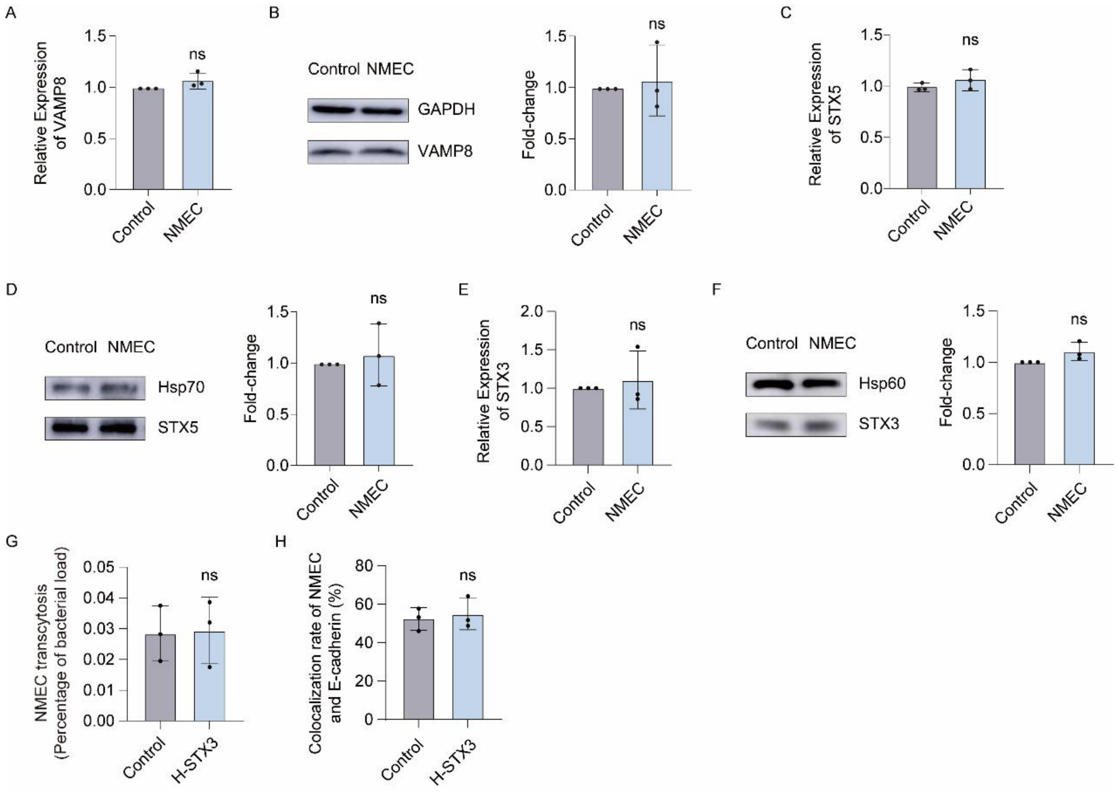

Fig. S5 NMEC increases its transcytosis efficiency by enhancing the expression of VAMP3 and STX4. (A, C, E) Analysis of VAMP8 (A), STX5 (C) or STX3 (E) expression in HBMECs and HBMECs infected by NMEC using qRT-PCR. (B, D, F) Representative western blotting image and quantitative analysis of VAMP8 (B), STX5 (D) or STX3 (F) in HBMECs and HBMECs infected by NMEC. GAPDH, loading control (B). Hsp70 or Hsp60, loading control (D and F). (G) Transcytosis of NMEC across H-STX3 cells compared with control cells. (H) Colocalization of intracellular NMEC with E-cadherin in polarized H-STX3 cells and control cells. The numbers indicate the percentage of BCVs colocalized with TfR relative to total intracellular BCVs (n = 3 slides). n = 3 independent experiments. Data represent the mean ± SD, ns, nonsignificant. Two-tailed unpaired Student's *t* test.

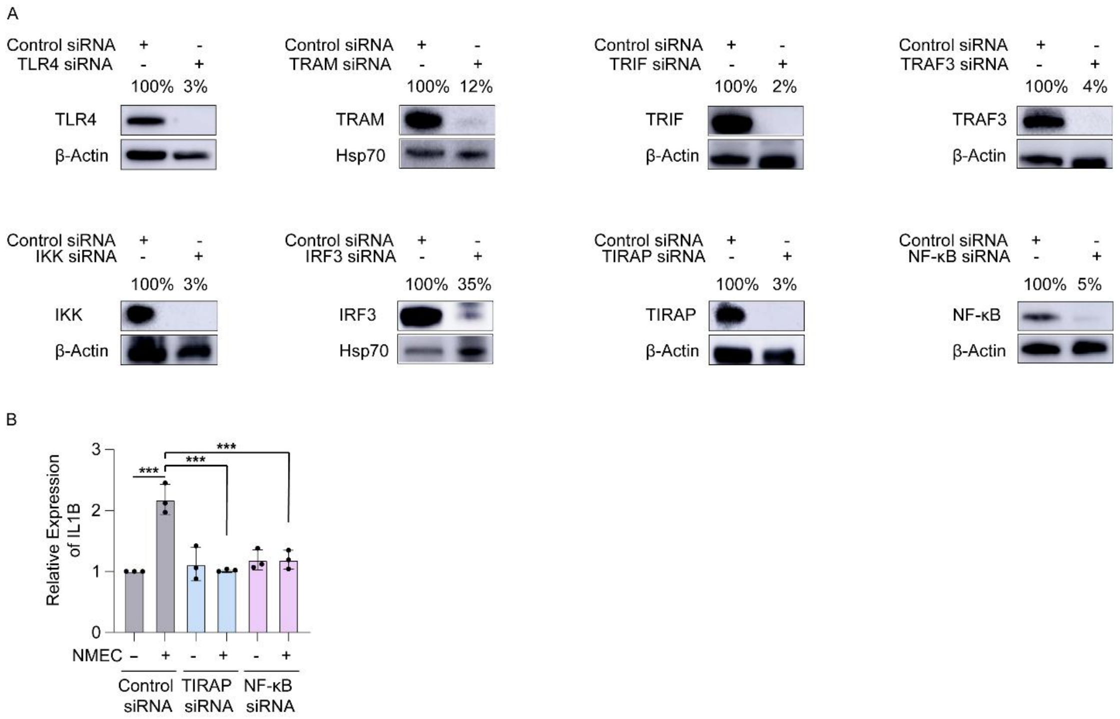

Fig. S6 The effect of silencing TLR4, TRAM, TRIF, TRAF3, IKK, IRF3, TIRAP and NF-κB in HBMECs. (A) HBMECs were transfected with siRNA targeting TLR4, TRAM, TRIF, TRAF3, IKK, IRF3, TIRAP, NF-κB or with control siRNA. The silencing efficiency is analyzed by immunoblotting. β-actin or Hsp70, loading control. (B) Analysis of IL1B expression in HBMECs transfected with control siRNA or siRNA targeting TIRAP or NF-κB in response to NMEC infection using qRT-PCR. n = 3 independent experiments. Data represent the mean ± SD, ns, nonsignificant. Two-way ANOVA.

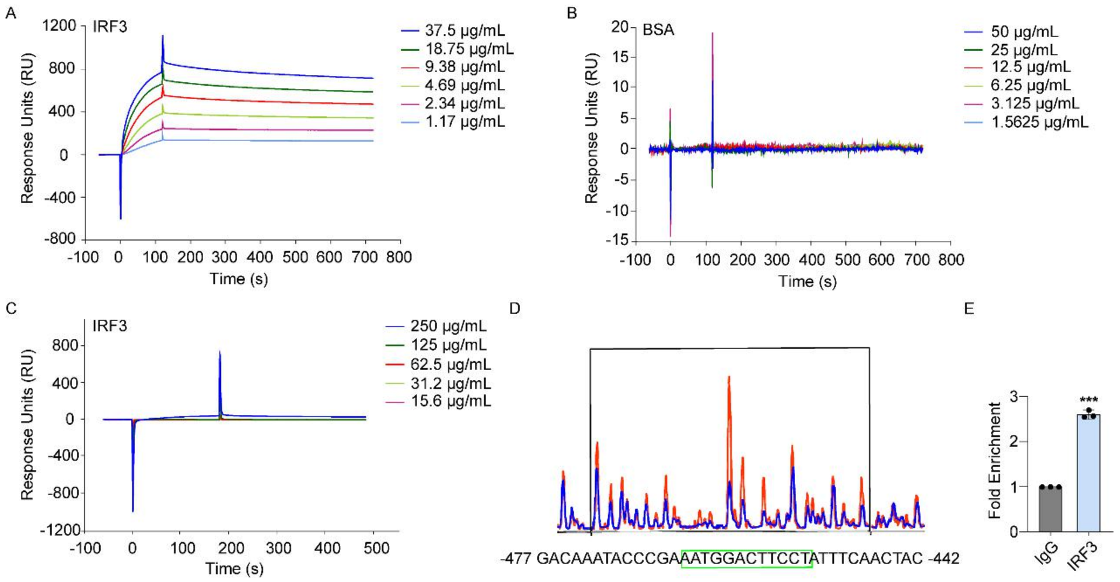

Fig. S7 IRF3 in the TLR4-TRIF-dependent pathway directly and indirectly regulates the expression of VAMP3 and syntaxin 4 respectively. (A-B) Binding affinities of IRF3 (A) or BSA (B) to the promoter of VAMP3 determined by an SPR assay. (C) Binding affinities of IRF3 to the promoter of STX4 determined by an SPR assay. (D) IRF3 binds to a motif in the promoter region of VAMP3. The protected region shows a significantly reduced peak intensities (blue) pattern than seen in compared with those of the control (red). The identified IRF3-binding motif is shown in a box at the bottom of the figure. (E) ChIP-qPCR analysis of the enrichment of the promoter region of VAMP3. IgG samples served as negative control. n = 3 independent experiments. Data are presented as the mean ± SD. * $P < 0.05$; ** $P < 0.01$; *** $P < 0.001$; ns represents no significance. Two-tailed unpaired Student's *t* test (E).

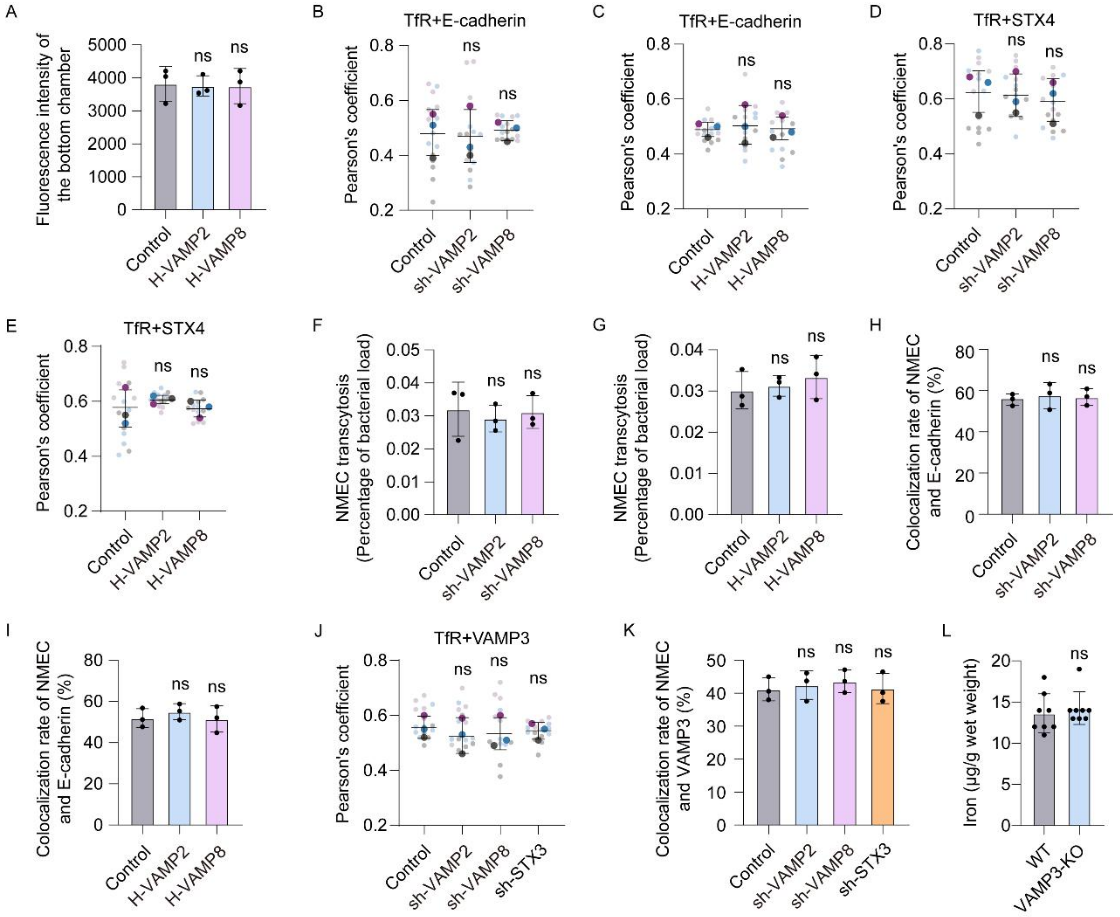

Fig. S8 VAMP2 and VAMP8 do not contribute to TfR transcytosis through HBMECs. (A) Spectrophotometric measurement of fluorescence intensity to assess FITC-Tf transcytosis from the apical side to the basolateral side of VAMP2-overexpressing (H-VAMP2) cells, VAMP8-overexpressing (H-VAMP8) cells or control cells. (B-E) Colocalization of TfR with E-cadherin (B, C) or STX4 (D, E) in polarized HBMECs transfected with the pGMLV-SC5 lentiviral vector harboring shRNA targeting VAMP2 (sh-VAMP2), VAMP8 (sh-VAMP8) and control shRNA (B, D) or H-VAMP2, H-VAMP8, and control cells (C, E). Quantification of colocalization was shown by calculating the Pearson correlation coefficients. (F, G) Transcytosis of NMEC across sh-VAMP2 cells and sh-VAMP8 cells (F), or H-VAMP2 cells and H-VAMP8 cells (G), compared with control cells. (H, I) Colocalization of

intracellular NMEC with E-cadherin in polarized sh-VAMP2 cells and sh-VAMP8 cells (H), or H-VAMP2 cells and H-VAMP8 cells (I), compared with control cells. The numbers indicate the percentage of BCVs colocalized with TfR relative to total intracellular BCVs (n = 3 slides). (J) Colocalization of VAMP3 with TfR in polarized sh-VAMP2 cells, sh-VAMP8 cells, sh-STX3 cells and control cells. Quantification of colocalization was shown by calculating the Pearson correlation coefficients. (K) Colocalization of intracellular NMEC with VAMP3 in polarized sh-VAMP2 cells, sh-VAMP8 cells, sh-STX3 cells compared with control cells. The numbers indicate the percentage of BCVs colocalized with TfR relative to total intracellular BCVs (n = 3 slides). n = 3 independent experiments. (L) Iron levels in the brains of VAMP3-deficient mice and wild-type mice. The IF data are quantified using Superplot (B-E and J), which concisely visualizes individual data points and their averages. The distinct combinations of colors indicate the three independent experiments performed. Horizontal lines show mean ± SD. Each small dot in the graph corresponds to a specific data point representing an analyzed image. The larger dots represent the average values calculated from the respective data points. Data are shown as the mean ± SD. ns, nonsignificant. One-way ANOVA (A-K), Two-tailed unpaired Student's *t* test (L).

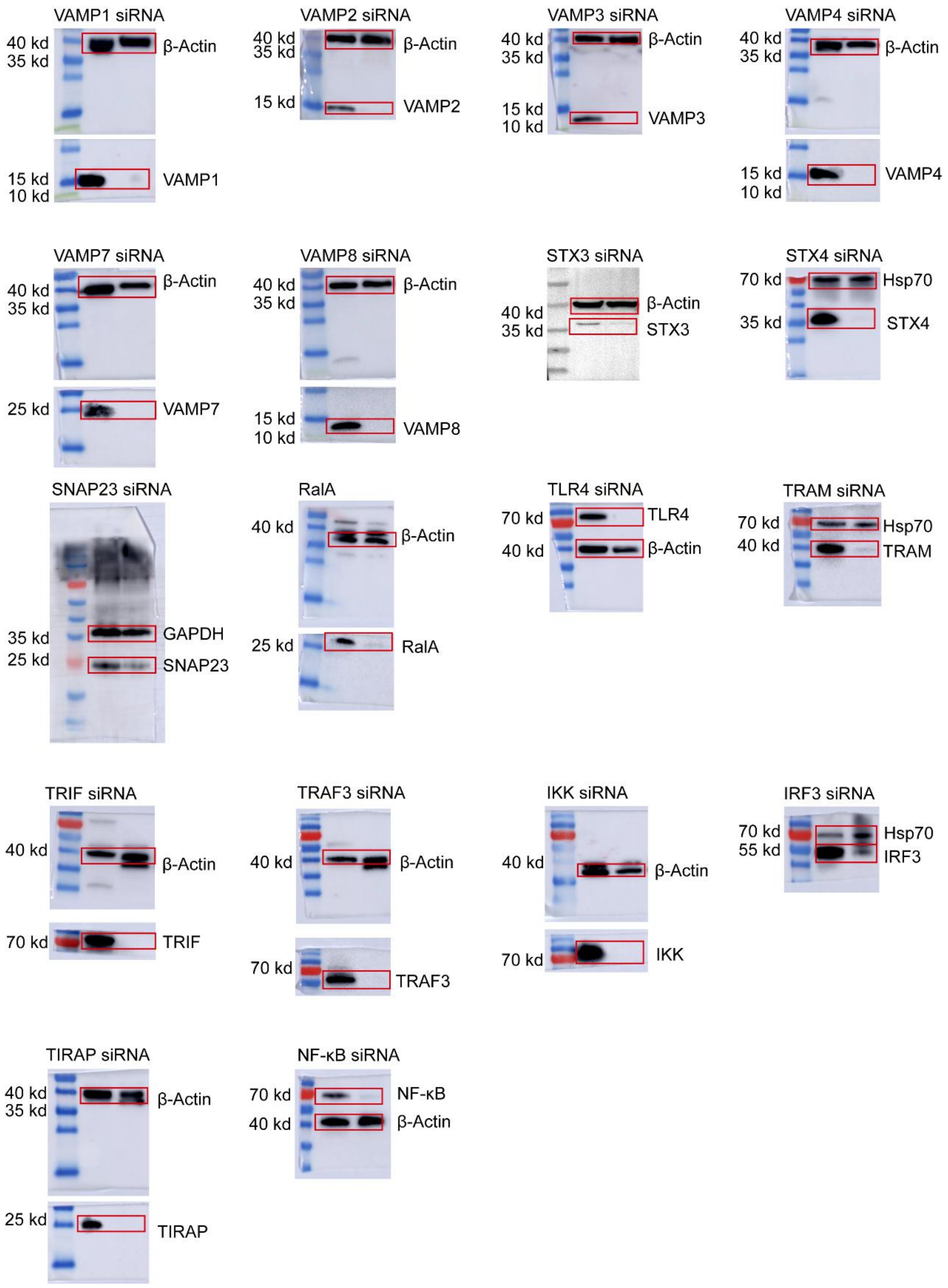

Fig. S9 Western blotting shows the efficiencies of siRNA-medicated knockdown at the protein level.

Supplementary Tables

***Table S1*. Primers used in this study.**

**Primer for Real-timePCR**		
**Target gene**	**F/R**	**Sequence (5'-3')**
VAMP3	F	ACAGGTCCAACTGCTGCCAC
VAMP3	R	GCGTCTGCACGGTCGTCTAA
VAMP8	F	CGTGTGCGGAACCTGCAAAG
VAMP8	R	TGAAACAGCTGGCCTCAGCC
syntaxin 4	F	CGGACAATTCGGCAGACTATT
syntaxin 4	R	TTCTGGGGCTCTATGGCCTT
syntaxin 5	F	AAACGCTACGGGTCTAAGAACA
syntaxin 5	R	GCACGCAAAGCTGGCTTATT
syntaxin 3	F	CGGCTTTTATGGACGAGTTCT
syntaxin 3	R	CTGCCGATGACCTGACCTC
IL1B	F	ATGATGGCTTATTACAGTGGCAA
IL1B	R	GTCGGAGATTCGTAGCTGGA
GAPDH	F	TGTGGGCATCAATGGATTTGG
GAPDH	R	ACACCATGTATTCCGGGTCAAT
**Primer for gene mutation**		
**Target gene**	**F/R**	**Sequence (5'-3')**
msbB	F	TATCATCAGTCGAAGGCCTCTCCTCGCGAGAGGCAT TTTGTGTAGGCTGGAGCTGCTTC
msbB	R	CACCAGATTGATTTTTGCCTTATCCGAAACTGGAAA AGCCATATGAATATCCTCCTTAG
**Primer for gene verification**		

Target gene	F/R	Sequence (5'-3')
msbB	F	GCTGAACCTGACTGCTCTC
msbB	R	GGAGTGAATACCACGACGAT
**Primer for gene complement construction**		
msbB	F	GCTCTAGATTATTTGATGGGATAAAGATCTTTGCGC
msbB	R	CCCAAGCTTTTAACATCCATTCGCAGCCGGTACGCAG
**Primer for gene complement identification**		
msbB	F	TTCAGAGCAAGAGATTACGC
msbB	R	CTAGCAGCACGCCATAGT
**Primer for DNase I footprinting**		
VAMP3	F	CAATGACCTATTAGTGTTTTATTTT
VAMP3	R	GGCGCGGCGCGGGGCAAA
syntaxin 4	F	TGGGGGGTGTGAGTGG
syntaxin 4	R	TTTCGAGGCAGTGGGTG
**siRNA or shRNA for gene knockdown**		
**Target gene**	**sense/anti-sense**	**Sequence (5'-3')**
VAMP1	sense	TGGACATCATACGTGTGAA
VAMP2	sense	GGCCTCCCAGTTTGAAACA
VAMP3	sense	GGACAAGGTTCTGGAAAGA
VAMP4	sense	GGAGAGACTAGATGAACTA
VAMP7	sense	CCAGACTACTTACGGTTCA
VAMP8	sense	GCAGAAGGTGGCTCGAAAA
syntaxin 3	sense	GGAACAAACUGAAGAGCAU
syntaxin 4	sense	CGACAGGCCTTAAATGAGA
TRAF3	sense	AGAGCATCGTTAAAGATAA
TLR4	sense	TGGTGAGTGTGACTATTGA

TRAM	sense	AAGCCACGGUGUGGAUACAAGUCAA
TRIF	sense	CCACCTCTCCAAATACCAA
IKKε	sense	GCATTGGAGTGACCTTGTA
IRF3	sense	AGAGGCTCGTGATGGTCAA
TIRAP	sense	GTGGCTTTCGTCAAGTCAA
SNAP23	sense	CCAACAGAGAUCGUAUUGATT

SI References

1. S. H. Huang *et al.*, Identification and characterization of an *Escherichia coli* invasion gene locus, ibeB, required for penetration of brain microvascular endothelial cells. *Infect. Immun.* **67**, 2103-2109 (1999).
2. K. A. Datsenko, B. L. Wanner, One-step inactivation of chromosomal genes in *Escherichia coli* K-12 using PCR products. *Proc. Natl. Acad. Sci. U. S. A* **97**, 6640-6645 (2000).
3. Y. J. Zuchero *et al.*, Discovery of Novel Blood-Brain Barrier Targets to Enhance Brain Uptake of Therapeutic Antibodies. *Neuron* **89**, 70-82 (2016).
4. Z. Cheng *et al.*, Pathogenic bacteria exploit transferrin receptor transcytosis to penetrate the blood-brain barrier. *Proc. Natl. Acad. Sci. U. S. A* **120**, e2307899120 (2023).
5. C. Guo, X. Yao, K. Wang, J. Wang, Y. Wang, Comparison of HIV-1 Gag and NCp7 in their selectivity for package signal, affinity for stem-loop 3, and Zn(2+) content. *Biochimie*. **179**, 135-145 (2020).
6. X. Li *et al.*, The Construction and Characterization of Mitochondrial Ferritin Overexpressing Mice. *Int. J. Mol. Sci.* **18**, 1518 (2017).
